# Knowledge Acquisition, Case Discussion, and Engagement in Health Professional Students Using Interactive Virtual Patient Cases Versus Written Case Studies: Randomized Controlled Trial

**DOI:** 10.2196/98700

**Published:** 2026-09-23

**Authors:** Jeffrey J Borckardt, Debra Henninger-Borckardt, Kelly Barth, Dusti Annan-Coultas, Lisa Langdale, Kimberly Kascak, Christopher Pelic, Michael A. de Arellano, Kathleen Brady

**Affiliations:** 1 Department of Psychiatry and Behavioral Sciences, College of Medicine, Medical University of South Carolina, 67 President Street, Charleston, SC, 29425, United States, 1 843-792-5807, 1 843-792-5702; 2 Palmetto Innovative Education, LLC, Mount Pleasant, SC, United States; 3 Office of Interprofessional Initiatives, Medical University of South Carolina, Charleston, SC, United States

**Keywords:** virtual patients, AI, large language models, ChatGPT, GPT-4o, interprofessional education, health professions education, simulation-based education, learner engagement, pain management education, randomized controlled trial

## Abstract

**Background:**

Effective pain and opioid management education remains a persistent challenge in health professions training. Static case studies and lecture-based instruction are widely used but may be insufficient to develop the clinical reasoning and communication skills required in practice. Interactive virtual patient simulations, including those powered by large language models, offer scalable alternatives, but head-to-head comparisons of interactive and static case formats using identical content among interprofessional learner populations remain limited.

**Objective:**

This randomized controlled trial examined whether learner knowledge, quiz performance, and engagement differed when educational case studies were delivered through an interactive, AI-enabled virtual patient format versus a static written format. Because the formats differ in interactivity as well as in their use of AI, the trial was designed to test the effect of an “interactive virtual patient format” rather than to isolate any AI-specific effect.

**Methods:**

Health professional students from medicine, nursing, dentistry, pharmacy, and allied health programs at a US academic health sciences university viewed a 12-minute video on pain management and were then randomized 1:1 to complete either 3 static written case studies or 3 interactive virtual patient case studies using the Figment Learning Labs Virtual Clinic. Case content was identical across conditions; only the delivery format was manipulated. The virtual patient system used an AI-enabled conversational interface (GPT-4o) delivering facilitator-authored case material, including a viewable medical record. Knowledge was assessed preintervention and postintervention using an 8-item test. Case discussion quiz accuracy and learner engagement (17 items) were also measured. Of 74 enrolled participants, 58 (78%) completed all study components and were included in this completer-based analysis. Analyses included a 2×2 mixed ANOVA and 2-tailed independent-sample *t* tests (α=.05).

**Results:**

A significant time (pre-post)×group (interactive vs written) interaction was observed (*F*_1,56_=4.39; *P*=.04; partial η²=0.07), indicating greater knowledge gains in the interactive condition. Participants in the virtual patient group demonstrated higher case discussion quiz accuracy (mean 0.79, SD 0.15) than those in the written case group (mean 0.64, SD 0.11; *t*_56_=4.48; *P*<.001). Agreement scores were significantly higher in the interactive group on 13 of 17 engagement items (*P*<.05), including items addressing realism, enjoyment, and perceived clinical relevance; because these were uncorrected item-level comparisons, they should be read as supportive rather than definitive.

**Conclusions:**

In this preliminary trial, exposure to interactive virtual patient cases was associated with higher engagement and higher short-term knowledge scores than static written cases. The design cannot isolate the independent contribution of AI, conversational interactivity, or time-on-task; the findings therefore support the value of an interactive virtual patient “format” rather than a distinct AI-specific advantage. Because the analysis was completer-based, featured unexplained attrition, and involved 2 authors who are founders of the company commercializing the platform, independent replication in larger, multi-institutional, and longitudinal samples is needed.

## Introduction

### Background

Effective pain management education, particularly regarding opioid risks, remains a challenge in health professions training. Despite initiatives such as the Pain Core Curriculum [1], critical knowledge gaps persist. Traditional approaches, including lectures, readings, and static case studies, often fall short of building the clinical reasoning skills required to address the complexities of patient-centered pain management [2]. Clinical rotations, while valuable, are constrained by availability, patient safety concerns, limited case exposure, and institutional barriers, leaving many students underprepared for nuanced decision-making in clinical practice [3,4].

Simulation-based learning (SBL) offers a solution, enabling practice, feedback, and skill development in complex encounters [5,6]. However, existing SBL formats involve distinct trade-offs in fidelity, cost, scalability, and standardization. Written cases lack interactivity [7], role-playing may feel artificial or inconsistent [8], laboratory-based simulations can be expensive [3], and standardized patient encounters, while realistic, are resource-intensive and limited in scope [9]. Textbox 1 summarizes the relative strengths and limitations of the major SBL modalities, situating interactive virtual patients within this landscape. Recent simulation research has emphasized the importance of deliberate practice, debriefing, fidelity alignment, cognitive scaffolding, and mastery progression to ensure that simulation produces meaningful learning gains [10-12]. There is a pressing need for innovative, scalable approaches that combine the benefits of simulation with greater efficiency and flexibility.

Textbox 1. **Relative strengths and limitations of major simulation-based learning modalities.**

**Written/static case studies**

- Strengths: Low cost, highly standardized, and easy to scale and distribute
- Limitations: No interactivity, limited fidelity, and do not require real-time data gathering or decision-making

**Role-play with peers**

- Strengths: Interactive and low cost
- Limitations: Variable realism and consistency, dependent on peers’ skills and engagement

**Manikin-/laboratory-based simulation**

- Strengths: High physical fidelity and hands-on procedural practice
- Limitations: High cost and infrastructure requirements, limited availability, and faculty-intensive

**Standardized (human) patients**

- Strengths: High interpersonal fidelity and realistic communication practice
- Limitations: Resource-intensive, limited scalability, and scheduling constraints

**Decision-tree virtual patients**:

- Strengths: Standardized, scalable, and interactive within preset branches
- Limitations: Constrained/branching dialogue and can feel scripted

**Large language model–based interactive virtual patients (this study)**

- Strengths: Scalable, open-ended natural-language interaction, real-time medical record and tool use, and asynchronous access
- Limitations: Potential for model errors/hallucinations, requires guardrails and authoritative source content, and dependent on the quality of case-authoring

Cost and scalability remain critical considerations in simulation. Procedural mastery experiences, while effective, can require substantial infrastructure and faculty investment. For example, mastery learning via virtual reality ultrasound simulation reached competency standards but required an estimated cost exceeding US $600 per trainee [10]. Other work in health professional education demonstrates that simulation-based mastery learning facilitates self-efficacy, authenticity of practice, and transfer to clinical performance when supported by repetition and cognitive frameworks [11].

The distinction between active and passive learning is an important confounder when comparing teaching and learning modalities. Multiple experimental studies demonstrate that interactive, problem-solving approaches improve learning outcomes and perceived learning more than passive, lecture-based instruction [13]. Students, particularly lower-achieving learners, benefit most from active engagement formats [13]. Earlier controlled trials similarly showed that fostering learner-to-learner interaction increased engagement and observable participation even when learning outcomes were equivalent [14]. Flipped classroom models likewise produce higher achievement, greater engagement, and improved retention compared to traditional didactic instruction [15], and gamified and game-based approaches have been associated with increased motivation and engagement in health professions learners [16]. These findings underscore the need to interpret comparative simulation studies through the lens of active-learning effects in addition to any technological capabilities.

### Virtual Patients and Large Language Models

Advances in digital simulation technologies and conversational interfaces have expanded opportunities for scalable, interactive learning environments. Virtual patient simulations can be implemented using a range of technologies, from structured decision-tree architectures to newer conversational systems supported by large language models (LLMs). These systems allow learners to engage in real-time dialogue with simulated patients and explore clinical reasoning processes in ways that more closely resemble clinical encounters [17-19]. LLM-driven clinical cases are increasingly being studied across a variety of educational scenarios [20], and this trial is distinguished by its head-to-head randomized comparison of interactive versus static delivery using identical case content in an interprofessional sample.

The pedagogical promise of LLM-based virtual patients must, however, be weighed against well-documented limitations of the underlying technology. LLMs generate responses probabilistically and can produce fluent but incorrect or fabricated content (“hallucinations”), exhibit pattern-driven or stereotyped responses, and vary across runs [21]. Recent work also indicates that straightforward, LLM-crafted cases are not always as engaging or as cognitively demanding as more complex, real-world cases [21]. These properties have motivated calls for explainable and verifiable AI in clinical and educational applications [22,23] and underscore the need for design safeguards, such as facilitator-authored ground-truth case content, explicit guardrails, and a priori scoring keys, when LLMs are used to deliver instructional material. We describe the specific safeguards used in this study in the “Methods” section.

### Aim and Hypotheses

Within this context, this randomized study examined whether learning outcomes differed when educational cases were delivered through interactive virtual patient encounters compared with static written case studies. We hypothesized that, relative to static written cases with identical content, the interactive virtual patient format would be associated with (1) greater pretraining to posttraining knowledge gains, (2) higher case discussion quiz accuracy, and (3) higher learner engagement among health professional students learning about pain and opioid management. Because the 2 formats differ in interactivity as well as in their use of AI, the study was explicitly designed to evaluate the “interactive virtual patient format” as a whole rather than to isolate the unique contribution of AI.

## Methods

### Study Design

This was a single-center, parallel-group, 1:1 randomized controlled trial comparing 2 case-delivery formats following a common didactic video. The trial is reported in accordance with the CONSORT-EHEALTH (Consolidated Standards of Reporting Trials of Electronic and Mobile Health Applications and Online Telehealth) checklist (Checklist 1).

### Participants

Participants were health professional students enrolled at a US-based academic health sciences university, representing programs in medicine, dentistry, nursing, pharmacy, and allied health. Inclusion criteria required active enrollment in clinical or preclinical coursework. An announcement about the study opportunity was made during an interprofessional course in the spring semester. Participation was voluntary.

### Ethics Approval

This study was approved by the Medical University of South Carolina’s Institutional Review Board for the Protection of Human Subjects (Pro00137370). All participants reviewed an online informed consent waiver prior to enrollment. No participant-identifying information was shared with the AI model or retained by the AI provider.

### Sample Size and Power

This was a preliminary (pilot-scale) trial intended to estimate effect sizes and establish feasibility; an a priori power analysis was not performed, and the achieved sample size was determined by voluntary enrollment within a single course offering. To contextualize the analyzed sample, a sensitivity analysis (computed in G*Power; Heinrich Heine University Düsseldorf) indicated that 58 completers yielded approximately 80% power at α=.05 to detect a between-within interaction in the medium-to-large range. Results are therefore interpreted as preliminary and hypothesis-generating, and the limitations of the sample size for generalizability are addressed in the “Discussion” section.

### Knowledge Assessment

A brief, custom 8-item multiple-choice test was developed by the investigators to assess performance on key learning objectives of the training activity: (1) distinguishing among pain types and definitions, (2) selecting evidence-based pain management strategies, (3) assessing opioid treatment considerations and risks, and (4) identifying disparities in pain management. Question stems were developed, and correct answers were determined by consensus of the investigative team; interrater reliability among the authors was not formally assessed. The test was administered pretraining and posttraining. A full copy of the knowledge assessment is included in Multimedia Appendix 1.

### Training Materials

For the didactic portion of this study, participants watched a 12-minute video lecture developed by the authors. The video covered (1) pain definitions, types, and etiologies; (2) empirically supported treatments for each pain type; (3) challenges associated with opioid prescribing identified in the empirical literature; (4) disparities in opioid prescribing; (5) prevalence of chronic pain by race and ethnicity; (6) factors influencing disparities in chronic pain prevalence; and (7) strategies for talking to patients about pain and opioids. All participants watched the same didactic video and were unable to skip any video content.

### Randomization and Case Conditions

Participants were randomized 1:1 by the software platform into 1 of 2 groups. Following the didactic video, participants completed either 3 written case studies or 3 interactive case studies. Case content was identical between groups; only the delivery method and the mode of interacting with the case material were manipulated (written cases vs virtual patient cases).

### Case Discussion Quiz Questions

In both groups, after completing each of the 3 case studies, participants answered three multiple-choice discussion questions: (1) Is this patient’s pain best categorized as nociceptive, neuropathic, both, or neither? (2) Is this patient’s pain acute, chronic, or neither? (3) Is this patient a good candidate for opioid therapy? Correct answers were determined a priori by the investigators; the same 3 questions were presented after each case, but correct answers varied as a function of case content. Participants received 1 point for each correct answer and could not return to the case information to answer the questions.

### Interactive Virtual Patients and Available Clinical Data

**Figure 1.**
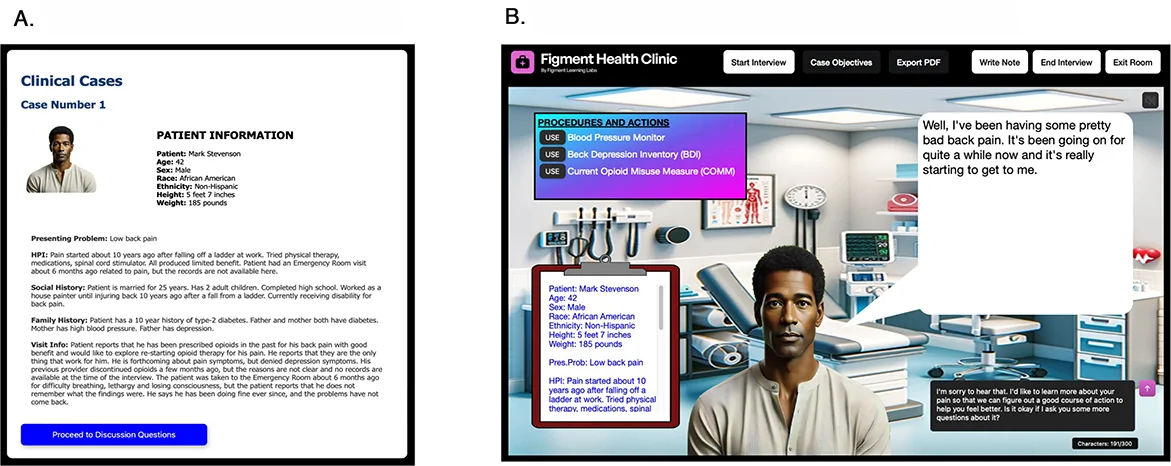
(A) Screenshot of the static, text-based case presentation format. (B) The Figment Learning Labs AI-powered interactive case interface used in this study.

The Figment Learning Labs Virtual Clinic is an online system that allows course facilitators to develop AI-powered interactive cases that students can interview. Importantly, the platform is not a chat-only interface: each case includes a viewable electronic medical record and a set of facilitator-defined assessments, tools, or procedures (eg, vital signs, laboratory results, imaging, medication and allergy lists, and prior treatments) that learners can access during the encounter. Virtual patient avatars (selectable by the facilitator) are animated against a clinic backdrop; the interface includes the patient’s medical record, an editable visit/progress note, and a full transcript available afterward for review (Figure 1).

Instructors specify learning objectives for each case along with patient information: name, age, sex, race, ethnicity, height, weight, personality, mood, communication style, chief complaint, history of present illness, social history, family and medical histories, medication list, allergies, and other relevant clinical information. Facilitators also indicate the procedures, tools, or assessments that learners can order, along with the corresponding results that populate the medical record. The record updates in real time when a tool is used, and the AI avatar is instructed on how to respond to each tool. A hidden facilitator instruction field provides more comprehensive case direction that is not visible to the learner. Interviews are online, asynchronous, and interactive, and they can be conducted via voice or text.

Because the clinical “facts” of each case (history, examination findings, laboratory and imaging results, medications, allergies, and the a priori correct discussion answers) are authored by the facilitator rather than generated de novo by the model, the case content available to learners includes the supporting clinical data needed for reasoning, and the determination of correct/incorrect reasoning does not depend on AI-generated facts.

### AI System Transparency, Accuracy, and Safeguards

At the beginning of a session, the facilitator-authored patient background information is sent to the OpenAI API as a system-level prompt, together with the activity context and explicit guardrail instructions that constrain the model to the role of the virtual patient and prevent it from supplying out-of-case clinical information or breaking character. Each learner utterance or tool use is relayed to the API, and the returned response is rendered as the virtual patient’s reply. The model also tracks a simulated emotional state, which drives the avatar’s facial expression so that learners can respond to verbal and nonverbal cues.

Several design features were intended to support clinical fidelity and reliability. First, the medical record, tool results, and correct discussion answers were fixed and facilitator-authored, so the substantive clinical content and scoring were independent of model variability. Second, guardrail prompts restricted the model to in-case information. Third, no learner-identifying information was shared with or retained by the provider; the API is a stateless instance that does not retain information after a session. We did not implement automated, turn-by-turn verification of every conversational response against the source record; the potential for occasional model errors (eg, hallucinations) and approaches to mitigate them are addressed as a limitation in the “Discussion” section.

### AI Model Information

Figment Clinic version 1.0 (Palmetto Innovative Education LLC, doing business as Figment Learning Labs) was used in conjunction with the OpenAI API and the ChatGPT (GPT-4o) model. The model parameters were as follows: temperature=0.7, top_*P*=0.9, frequency_penalty=0.8, presence_penalty=0.8, and max_tokens=500.

### Case Study Information

The 3 cases were developed by the authors to represent common encounters with patients presenting with acute, chronic, neuropathic, and nociceptive pain, and each specified the patient’s demographic and social context (including age, sex, race, ethnicity, and relevant social history) so that learners encountered contextualized rather than generic patients. All participants received the same 3 cases; only presentation format (written vs interactive) was randomized. Case study details are provided in Multimedia Appendix 1.

### Learner Engagement Survey

After completing all training and knowledge tests, participants completed an online engagement survey developed by the authors, consisting of 17 items assessing interface usability and value, engagement and satisfaction, and applicability of the information to real-world scenarios. Items used a 5-point Likert scale (1=“strongly disagree” to 5=“strongly agree”).

### Procedures

Study enrollment opened on April 1, 2025, and closed on July 31, 2025. Participants visited the study website, reviewed the informed consent waiver online, completed a demographics form and a baseline knowledge assessment, and were then randomized in a 1:1 ratio by the online system to either written or interactive cases. All participants then viewed the 12-minute educational video on evidence-based pain and opioid management, with an emphasis on care for diverse populations. In the written condition, participants reviewed 3 text vignettes (each with a patient photograph) and answered the discussion questions. In the interactive condition, the same cases were presented as simulated telehealth consultations with AI-powered virtual patients, and participants conducted online interviews to elicit case details (rather than only reading the material in text format) before answering the same discussion questions. After the learning activity, all participants repeated the knowledge test and completed the engagement survey. All sessions were conducted remotely and asynchronously and lasted approximately 45 to 60 minutes.

### Statistical Analysis

Knowledge gains were assessed via a 2×2 mixed ANOVA examining knowledge test scores (percent correct) pretraining and posttraining and between groups (written vs interactive). Case discussion accuracy and learner self-report ratings were compared between conditions using 2-tailed independent-sample *t* tests. Where Levene test indicated unequal variances, the Welch correction was applied and the adjusted degrees of freedom were reported. Internal consistency for each instrument was estimated with Cronbach α. Analyses were conducted in SPSS (version 27; IBM Corp), with the critical α set at .05. Because the 17 engagement items were analyzed individually without correction for multiple comparisons, item-level results are interpreted as supportive rather than confirmatory.

## Results

### Participants and Attrition

**Figure 2.**
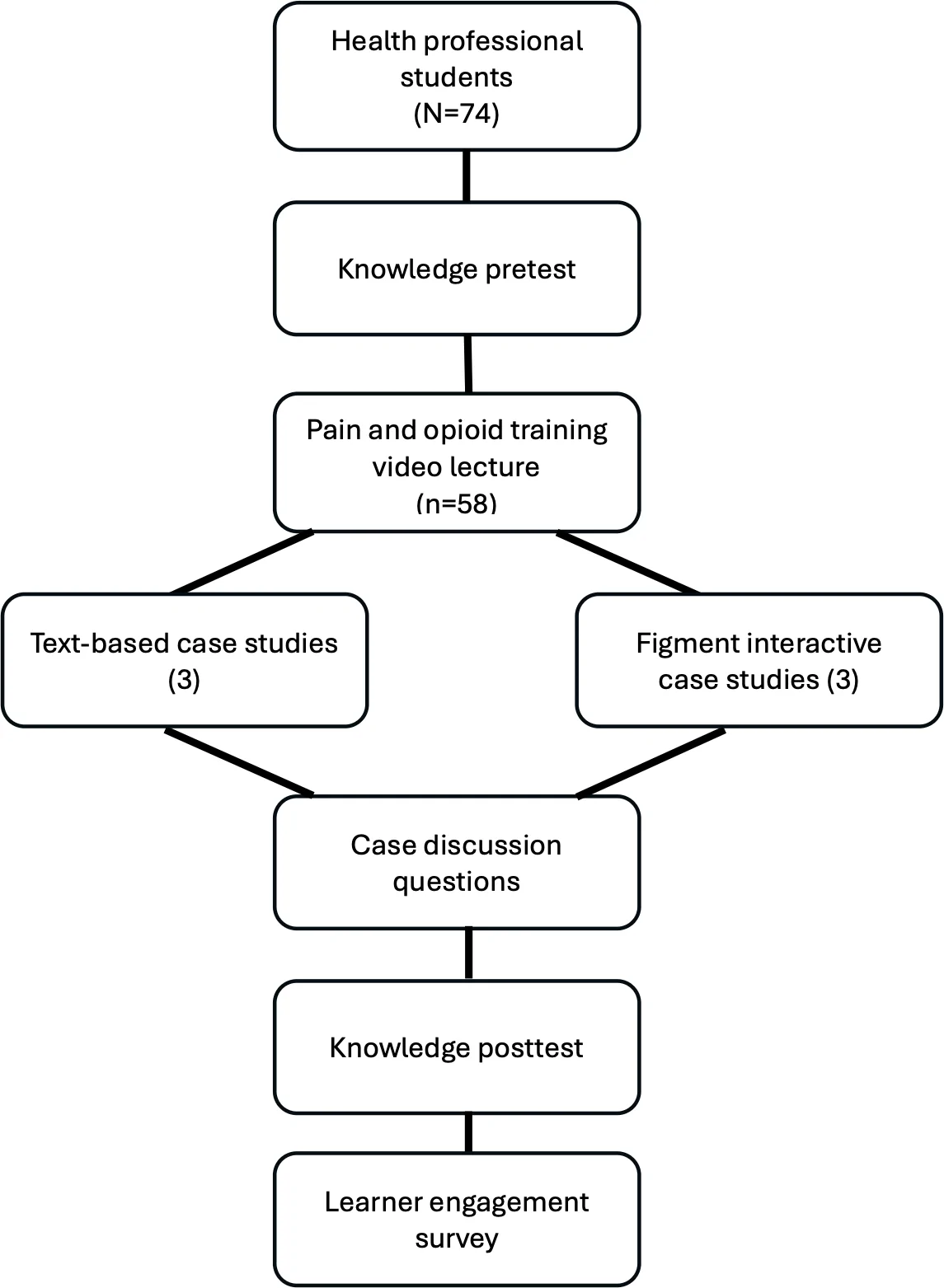
Study procedure diagram.

A total of 74 participants were enrolled, and 58 (78%) completed all study components and were included in the completer-based analysis. All 16 noncompleters (22%) discontinued before finishing the video training and did not begin the case study portion; reasons for discontinuation were not collected and are unknown. Baseline knowledge test data were available for 63 of the 74 enrolled participants (all 58 completers and 5 of the 16 noncompleters); the degrees of freedom for the baseline comparisons in Table 1 reflect this denominator. There were no significant differences between completers and noncompleters in sex, race, ethnicity, degree program, or age (Table 1), but because the reasons for attrition are unknown, the possibility that unmeasured differences biased the observed group differences cannot be excluded (see “Limitations” section). The overall study procedures are summarized in Figure 2.

**Table 1.** Breakdown of sample sizes (n), mean age, and mean pretest scores by participant sex, race, ethnicity, degree program, group (written cases vs interactive virtual patient cases), and status (study completers vs noncompleters).

Characteristics	Written cases^a^	Interactive cases	Significance		Completers	Noncompleters	Significance		Pretest score, mean (SD)^b^	Significance^c^		Total
			Statistic	*P* value			Statistic	*P* value		Statistic	*P* value	
Age (y), mean (SD)	27.16 (8.34)	27.44 (8.10)	0.15 (72)^d^	.88	26.07 (6.74)	31.75 (11.19)	1.94 (72)^d^	.07	27.30 (8.17)	—^e^		27.30 (8.17)
Sex, n			2.06 (1)^f^	.15			0.08 (1)^f^	.77		0.96 (61)^d^	.34	
Male	8	13			16	5			5.11 (1.02)			21
Female	30	23			42	11			5.42 (1.22)			53
Race, n			1.95 (3)^f^	.58			2.46 (3)^f^	.48		1.80 (3, 59)^g^	.16	
White	26	26			42	10			5.44 (1.16)			52
Asian	7	7			9	5			5.50 (.97)			14
Black	3	3			5	1			4.67 (1.21)			6
Multiracial	2	0			2	0			4.00 (1.41)			2
Ethnicity			1.19 (2)^f^	.55			2.47 (2)^f^	.29		0.75 (2, 60)^g^	.48	
Hispanic	1	3			4	0			5.00 (.82)			4
Non-Hispanic	35	31			50	16			5.40 (1.13)			66
Prefer not to say	2	2			4	0			4.75 (1.89)			4
Group			—	—			0.47 (1)^f^	.49		1.61 (61)^d^	.11	
Written cases	—	—			31	7			5.10 (1.14)			38
Interactive cases	—	—			27	9			5.56 (1.16)			36
Status			0.47 (1)^f^	.49			—	—		0.93 (61)^d^	.35	
Completers	31	27			—	—			5.29 (1.17)			58
Noncompleters	7	9			—	—			5.80 (1.01)			16
Degree			3.84 (5)^f^	.57			8.86 (5)	.12		1.08 (5, 57)^g^	.38	
Medicine	9	10			13	6			5.08 (1.04)			19
Dental medicine	1	0			1	0			6.00 (SD not estimable, n=1)			1
Pharmacy	2	6			7	1			4.57 (1.27)			8
Allied health professions	22	17			34	5			5.56 (1.12)			39
Graduate studies	3	2			2	3			5.40 (1.34)			5
Nursing	1	1			1	1			5.00 (SD not estimable, n=1)			2

a All 74 enrolled participants are represented (58 completers and 16 noncompleters). Group columns show the allocation of all enrolled participants, while the completers and noncompleters columns partition that same sample.

b Baseline (pretest) knowledge scores were available for 63 of the 74 enrolled participants (all 58 completers and 5 of the 16 noncompleters). The *df* for the baseline comparisons reflect this denominator.

c Comparisons of categorical variables were conducted using chi‑square tests of independence. Comparisons of age and baseline knowledge scores by sex, group, and completion status were performed using independent‑sample *t* tests. Comparisons of baseline knowledge scores across race, ethnicity, and degree program were conducted using one‑way ANOVA.

d Value is reported as *t* test (*df*).

e N/A: not available.

f Value is reported as chi-square (*df*).

g Value is reported as *F* test (*df*).

Among the 58 completers, 16 (28%) were male and 42 (72%) were female; mean age was 26.07 (SD 6.74) years. Self-reported race was White (n=42, 72%), Asian (n=9, 16%), Black (n=5, 9%), and multiracial (n=2, 3%); ethnicity was Hispanic (n=4, 7%), non-Hispanic (n=50, 86%), and prefer-not-to-say (n=4, 7%). Degree programs were allied health professions (n=34, 59%), medicine (n=13, 22%), pharmacy (n=7, 12%), graduate studies (n=2, 3%), dental medicine (n=1, 2%), and nursing (n=1, 2%). Of the 74 enrolled participants, 38 (51%) were randomized to the written-case group and 36 (49%) to the interactive-case group; among the 58 completers, 31 (53%) were in the written-case group and 27 (47%) in the interactive-case group. Sample sizes, mean age, and mean pretest knowledge scores by category are shown in Table 1.

### Knowledge Gains

**Figure 3.**
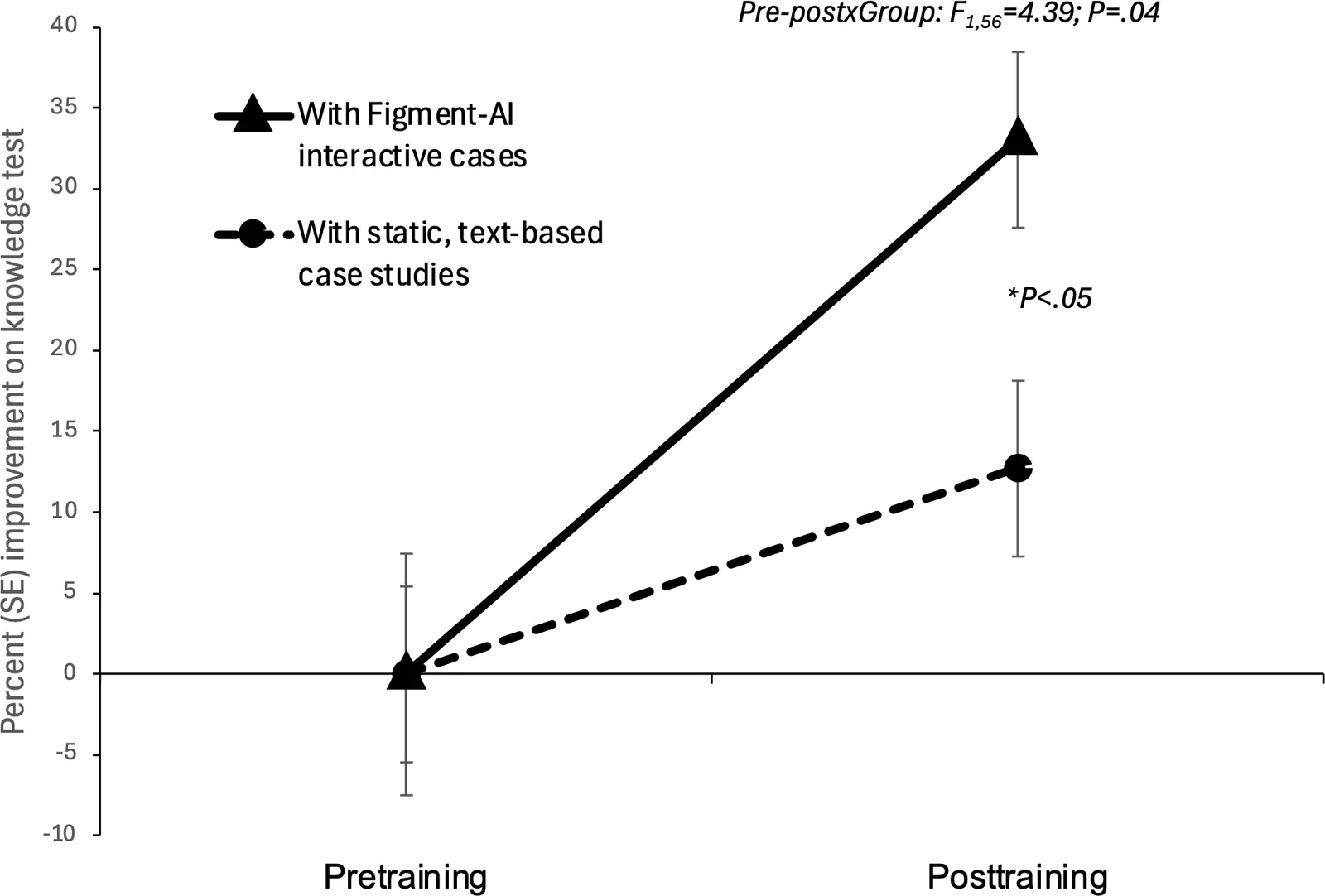
Mixed ANOVA results showing changes in knowledge scores from pretraining to posttraining by group (interactive vs static).

Internal consistency of the 8-item knowledge test was modest (Cronbach α=0.56), as expected for a brief, custom instrument, and this value should be considered when interpreting the knowledge results. A 2×2 mixed ANOVA revealed a significant time (pretraining to posttraining)×group (written vs interactive) interaction (*F*_1,56_=4.39; *P*=.04; partial η²=0.07), indicating greater knowledge gains in the interactive condition; the posttest between-group difference was also significant (*P*=.03). Figure 3 shows the percent improvement from pretraining to posttraining by group.

### Case Discussion Quiz Accuracy

Internal consistency of the case discussion measure across the 9 dichotomously scored items (3 discussion questions after each of 3 cases) was low (Cronbach α=0.31). This is expected and does not necessarily indicate measurement error: although the same prompts followed each case, the correct responses differed across cases, which represented distinct clinical scenarios. The items were not intended to function as parallel indicators of a single latent construct; rather, they sampled performance across heterogeneous cases. Accordingly, the case discussion score is interpreted as an aggregate performance measure across cases rather than as a homogeneous psychometric scale. Participants in the interactive condition demonstrated significantly higher accuracy (mean 0.79, SD 0.15) than those in the written condition (mean 0.64, SD 0.11; *t*_56_=4.48; *P*<.001), corresponding to an approximately 25% relative advantage for the interactive format.

### Learner Engagement

**Figure 4.**
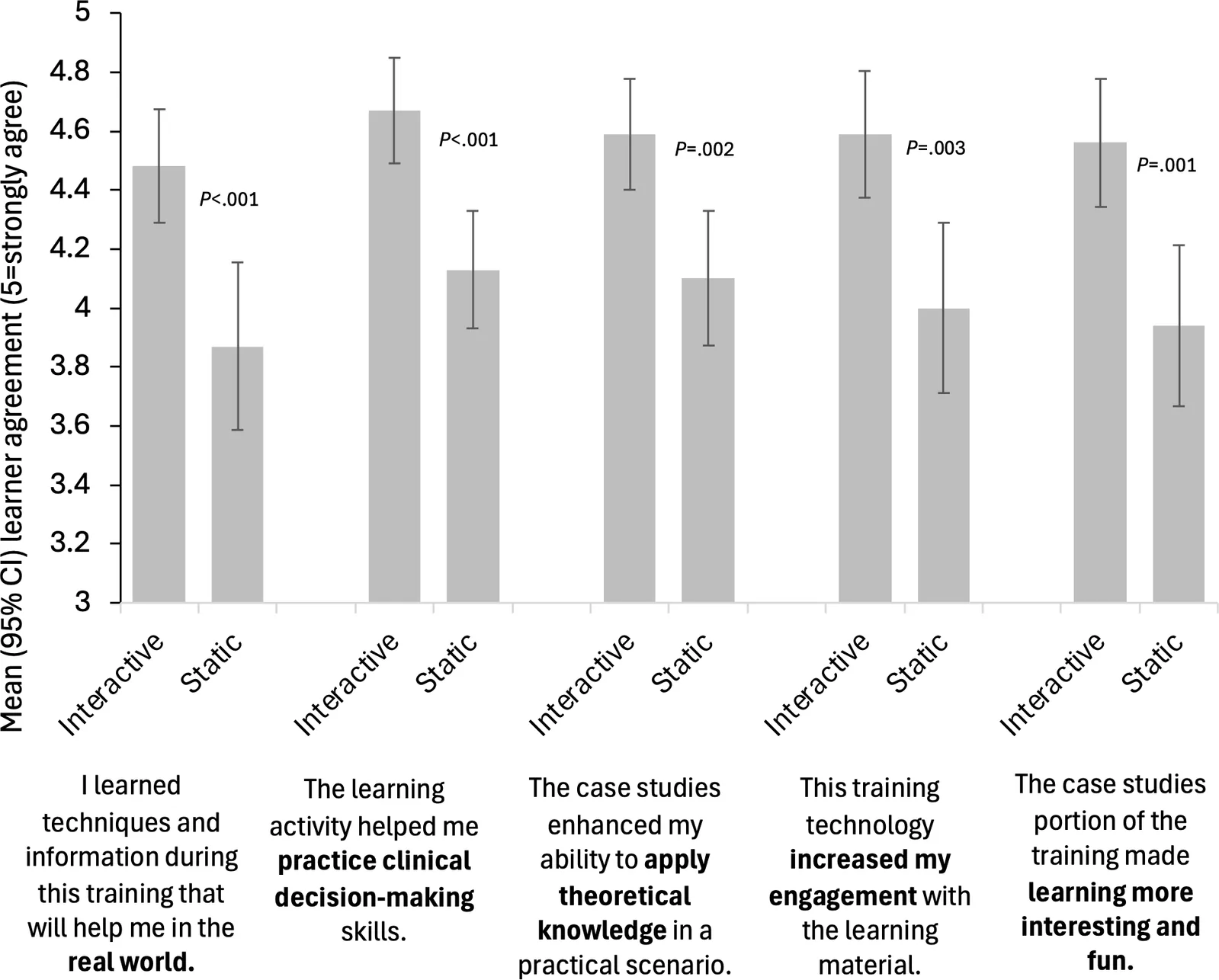
Learner mean performance (95% CI) on key engagement survey items (interactive vs static).

Internal consistency of the 17-item engagement questionnaire was high (Cronbach α=0.95). Agreement scores were significantly higher in the interactive group than in the written group on 13 of the 17 items (*P*<.05). Figure 4 shows group-level means (95% CI) on five key indicator items: (1) I learned techniques and information during this training that will help me in the real world; (2) the learning activity helped me practice clinical decision-making skills; (3) the case studies enhanced my ability to apply theoretical knowledge in a practical scenario; (4) this training technology increased my engagement with the learning material; and (5) the case-studies portion made learning more interesting and fun. Full item-level results are shown in Table 2. Because these were 17 uncorrected, item-level comparisons, the pattern is interpreted as consistently supportive of greater engagement in the interactive condition rather than as a set of independent confirmatory findings.

**Table 2.** Mean Likert rating score^a^ for learner engagement items among participants in the interactive virtual patient case group compared with those in the written case group^b^.

Learner engagement survey item and group	Number, n	Mean (SD)	*t* test (*df*)	*P* value
The online training interface was easy to use.			1.75 (55.92)	.09
Interactive cases	27	4.56 (0.51)		
Written cases	31	4.29 (0.78)		
The instructions for completing the online training were clear and understandable.			0.77 (55.92)	.45
Interactive cases	27	4.41 (0.57)		
Written cases	31	4.29 (0.59)		
The online training platform is technologically sophisticated.			3.81 (47.69)	<.001
Interactive cases	27	4.48 (0.51)		
Written cases	31	3.74 (0.93)		
The online training platform is visually appealing.			3.46 (56)	.001
Interactive cases	27	4.52 (0.51)		
Written cases	31	3.74 (1.06)		
The case studies portion of the learning activity met the learning objectives.			2.33 (56)	.02
Interactive cases	27	4.52 (0.58)		
Written cases	31	4.16 (0.58)		
The case studies felt like “real-world” scenarios.			2.00 (55.36)	.052
Interactive cases	27	4.63 (0.56)		
Written cases	31	4.32 (0.60)		
The case studies portion of the training made learning more interesting and fun.			3.42 (56)	.001
Interactive cases	27	4.56 (0.58)		
Written cases	31	3.94 (0.77)		
I felt that I learned something from the case studies.			3.89 (56)	<.001
Interactive cases	27	4.59 (0.57)		
Written cases	31	4.00 (0.78)		
I learned techniques and information during this training that will help me in the real world.			3.39 (56)	.001
Interactive cases	27	4.48 (0.51)		
Written cases	31	3.87 (0.81)		
The learning activity helped me practice clinical decision-making skills.			3.88 (56)	<.001
Interactive cases	27	4.67 (0.48)		
Written cases	31	4.13 (0.56)		
The case studies enhanced my ability to apply theoretical knowledge in a practical scenario.			3.88 (55.90)	<.001
Interactive cases	27	4.59 (0.50)		
Written cases	31	4.10 (0.65)		
The case studies felt realistic.			1.74 (56)	.09
Interactive cases	27	4.41 (0.80)		
Written cases	31	4.03 (0.84)		
This training technology increased my engagement with the learning material.			3.15 (56)	.002
Interactive cases	27	4.59 (0.57)		
Written cases	31	4.00 (0.82)		
I am motivated to use activities like online case studies to enhance my learning experience.			3.30 (56)	.002
Interactive cases	27	4.56 (0.51)		
Written cases	31	3.97 (0.80)		
This training activity made learning more enjoyable.			3.47 (56)	.001
Interactive cases	27	4.52 (0.51)		
Written cases	31	3.90 (0.79)		
I am satisfied with this learning activity.			3.53 (56)	.001
Interactive cases	27	4.52 (0.51)		
Written cases	31	3.97 (0.66)		
I would recommend this learning activity to my peers.			4.56 (56)	<.001
Interactive cases	27	4.56 (0.51)		
Written cases	31	3.84 (0.69)		

a Items used a 5-point Likert scale (1=“strongly disagree” to 5=“strongly agree”).

b All comparisons were conducted using 2-tailed independent-sample *t* tests (interactive cases, n=27; written cases, n=31). Where Levene test indicated unequal variances, the Welch correction was applied, and the adjusted *df* are reported.

## Discussion

### Principal Findings

In this randomized controlled trial, students assigned to the interactive case condition demonstrated higher short-term knowledge gains, higher case discussion accuracy, and higher engagement than students assigned to the written case condition with identical content. These findings support the value of an interactive virtual patient format for case-based learning in health professions education. They are, however, preliminary and primarily hypothesis-generating, and, as detailed below, the design does not permit attribution of the observed benefits to AI specifically.

### Interpretation: An Interactive-Format Effect, Not an Isolated AI Effect

The strongest interpretation supported by this design is that an interactive, AI-enabled virtual patient format outperformed a static written format delivering the same content. The trial cannot separate the contribution of AI from the contributions of conversational interactivity, learner agency, adaptive questioning, or additional time-on-task because these features are bundled within the interactive condition. We therefore frame the results as support for interactive virtual patient learning rather than as evidence of a distinct, AI-specific advantage. At a minimum, integrating an LLM did not detract from the benefits of interactive learning; quantifying the incremental value of AI over non-AI interactive formats will require trials that include a non-AI interactive comparison arm.

### Comparison With Prior Work

Our findings align with and extend prior research on simulation-based and active learning, adding randomized evidence that an interactive virtual patient platform can yield both measurable learning advantages and operational scalability [5,6,10-12]. The use of AI to deliver clinical cases has been examined in a range of recent studies [20], and some work suggests that simple LLM-generated cases may be less engaging than complex real-world cases [21]; our results, using facilitator-authored cases delivered through an interactive interface, are consistent with the broader active-learning literature [13-15] showing the benefits of interactivity over passive formats. Although this study addressed pain management, the instructional mechanism is not necessarily content-specific, and the observed benefits may extend to other domains that rely on clinical reasoning, communication, and applied decision-making. The extent to which these effects generalize across content areas, learner levels, and curricular structures remains an empirical question.

### Clinical Reasoning, Data Access, and Demographic Context

Interactive virtual patients can train skills beyond communication; in the platform used here, learners could access a structured medical record and order facilitator-defined assessments (eg, laboratory results, medication and allergy lists, and prior treatments), so the encounters were designed to *unfold* components of case information (as opposed to openly presenting them as part of the vignette, as in the text-based case format) in addition to facilitating conversation alone. Nonetheless, this study did not separately measure information-gathering behavior, and future work should evaluate how access to and use of clinical data within the simulation relate to reasoning outcomes. Second, the realism of any virtual patient depends on the demographic and socioeconomic context authored into the case. The 3 cases here specified patient demographics and social context, but 3 US-based cases cannot represent the full range of geographic, socioeconomic, and cultural circumstances that shape pain management; readers should not generalize these specific encounters to all settings, and future case libraries should deliberately span diverse contexts and be evaluated for bias.

### AI Reliability and the Need for Verifiable Content

LLMs can generate fluent but incorrect content, exhibit stereotyped patterns, and vary across runs [21]. In this study, the substantive clinical content and the scoring of reasoning were fixed and facilitator-authored, and guardrail prompts constrained the model to in-case information, which limits, but does not eliminate, the risk that a conversational reply could be inaccurate. We did not perform automated, turn-by-turn verification of model responses against the source record. Educational deployments of LLM-based virtual patients should therefore incorporate safeguards such as authoritative source content, explicit guardrails, human review, and, where feasible, explainable or verification-oriented architectures [22,23], and they should also monitor for inaccurate or biased output.

### Limitations

Several limitations should be acknowledged. First, this was a completer-based analysis: 16 of 74 enrolled participants (22%) discontinued before the case study portion, and because reasons for attrition are unknown, unmeasured differences between completers and noncompleters could have biased the observed group differences and may limit conclusions about feasibility, acceptability, and generalizability. Second, time-on-task was not measured; students in the interactive condition may have spent more time with the material, and this duration effect cannot be disentangled from the modality itself. Third, voluntary participation may introduce self-selection bias toward learners more favorably disposed toward technology-assisted learning. Fourth, the assessment instruments were brief, with modest (knowledge test; Cronbach α=0.56) to low (aggregate discussion score; Cronbach α=0.31) internal consistency, limiting the granularity of measured learning. Fifth, the study was conducted at a single center with a modest sample (n=58) and was powered only to detect medium-to-large effects; small effects could have been missed. Sixth, only immediate postintervention outcomes were assessed; data on knowledge and skill retention at 6 or 12 months were not collected and are an important gap. Finally, the effects of AI cannot be separated from the broader benefits of interactivity, and 2 authors (JJB and DH) are founders of the company that commercializes the platform under study; although this conflict is disclosed and the substantive content and scoring were author-fixed, independent replication by investigators without a commercial interest is essential.

### Future Directions

Future research should include a non-AI interactive comparison arm to isolate any incremental AI effect; measure time-on-task and in-simulation data-gathering behavior; assess retention at 6 and 12 months and transfer to authentic clinical performance; use validated, multifaceted assessments; and recruit larger, multi-institutional, and demographically diverse samples through curricular integration. Work on explainable and verifiable AI [22,23] may help ensure that conversational responses remain clinically accurate and interpretable as these tools scale.

### Conclusions

This preliminary randomized controlled trial suggests that an interactive virtual patient format may be a portable, scalable, and feasible way to realize the benefits of interactive, case-based learning. Compared with static written cases of identical content, students in the interactive condition showed greater short-term knowledge gains, higher case discussion accuracy, and stronger engagement. These results are hypothesis-generating and reflect an interactive-format effect that cannot be attributed to AI specifically; they should be interpreted in light of completer-based analysis with unexplained attrition, voluntary participation, brief assessments, the absence of time-on-task and retention data, and the authors’ commercial interest. Larger, multi-institutional, longitudinal, and independently conducted trials—ideally including non-AI interactive comparison conditions—are needed to confirm durability and generalizability and to determine the unique contribution, if any, of AI to learning outcomes.

## Supplementary material

10.2196/98700 Multimedia Appendix 1 Knowledge test items and case study information.

10.2196/98700 Checklist 1 CONSORT-EHEALTH (V 1.6.1) checklist.

## Abbreviations

CONSORT-EHEALTH: Consolidated Standards of Reporting Trials of Electronic and Mobile Health Applications and Online Telehealth
LLM: large language model
SBL: simulation-based learning
