# Supplementary material for: Knowledge Acquisition, Case Discussion, and Engagement in Health Professional Students Using Interactive Virtual Patient Cases Versus Written Case Studies: Randomized Controlled Trial
Source: JMIR Nurs. 2026 Sep 23;9:e98700. doi: 10.2196/98700 (PMC13600594; doi:10.2196/98700)
Supplement: Supplementary file 1 [file nursing-v9-e98700-s002.pdf]

CONSORT-EHEALTH (V 1.6.1) - Submission/Publication Form

The CONSORT-EHEALTH checklist is intended for authors of randomized trials evaluating web-based and Internet-based applications/interventions, including mobile interventions, electronic games (incl multiplayer games), social media, certain telehealth applications, and other interactive and/or networked electronic applications. Some of the items (e.g. all subitems under item 5 - description of the intervention) may also be applicable for other study designs.

The goal of the CONSORT EHEALTH checklist and guideline is to be
a) a guide for reporting for authors of RCTs,
b) to form a basis for appraisal of an ehealth trial (in terms of validity)

CONSORT-EHEALTH items/subitems are MANDATORY reporting items for studies published in the Journal of Medical Internet Research and other journals / scientific societies endorsing the checklist.

Items numbered 1., 2., 3., 4a., 4b etc are original CONSORT or CONSORT-NPT (non-pharmacologic treatment) items.
Items with Roman numerals (i., ii, iii, iv etc.) are CONSORT-EHEALTH extensions/clarifications.

As the CONSORT-EHEALTH checklist is still considered in a formative stage, we would ask that you also RATE ON A SCALE OF 1-5 how important/useful you feel each item is FOR THE PURPOSE OF THE CHECKLIST and reporting guideline (optional).

Mandatory reporting items are marked with a red *.
In the textboxes, either copy & paste the relevant sections from your manuscript into this form - please include any quotes from your manuscript in QUOTATION MARKS,
or answer directly by providing additional information not in the manuscript, or elaborating on why the item was not relevant for this study.

YOUR ANSWERS WILL BE PUBLISHED AS A SUPPLEMENTARY FILE TO YOUR PUBLICATION IN JMIR AND ARE CONSIDERED PART OF YOUR PUBLICATION (IF ACCEPTED).
Please fill in these questions diligently. Information will not be copyedited, so please use proper spelling and grammar, use correct capitalization, and avoid abbreviations.

DO NOT FORGET TO SAVE AS PDF _AND_ CLICK THE SUBMIT BUTTON SO YOUR ANSWERS ARE IN OUR DATABASE !!!

Citation Suggestion (if you append the pdf as Appendix we suggest to cite this paper in the caption):
Eysenbach G, CONSORT-EHEALTH Group
CONSORT-EHEALTH: Improving and Standardizing Evaluation Reports of Web-based and Mobile Health Interventions
J Med Internet Res 2011;13(4):e126
URL: http://www.jmir.org/2011/4/e126/
doi: 10.2196/jmir.1923
PMID: 22209829

borckard@gmail.com Switch account

Not shared

Draft saved

* Indicates required question

Your name *

First Last

Jeffrey Borckardt

Primary Affiliation (short), City, Country *

University of Toronto, Toronto, Canada

Medical University of South Carolina

Your e-mail address *

abc@gmail.com

borckard@musc.edu

Title of your manuscript *

Provide the (draft) title of your manuscript.

Interactive Virtual Patient Cases Versus Written Case Studies: A Randomized Controlled Trial of Knowledge Acquisition, Case Discussion Performance, and Engagement in Health Professional Students

Name of your App/Software/Intervention *

If there is a short and a long/alternate name, write the short name first and add the long name in brackets.

Figment Clinic (Figment Learning Labs)

Evaluated Version (if any)

e.g. "V1", "Release 2017-03-01", "Version 2.0.27913"

V1.0 Release 2025-01-01

Language(s) *

What language is the intervention/app in? If multiple languages are available, separate by comma (e.g. "English, French")

English

URL of your Intervention Website or App

e.g. a direct link to the mobile app on app in appstore (itunes, Google Play), or URL of the website. If the intervention is a DVD or hardware, you can also link to an Amazon page.

FigmentHealth.com

URL of an image/screenshot (optional)

https://figmentprograms.com/Screenshot.png

Accessibility *

Can an enduser access the intervention presently?

- ○ access is free and open
- ○ access only for special usergroups, not open
- ◉ access is open to everyone, but requires payment/subscription/in-app purchases
- ○ app/intervention no longer accessible
- ○ Other:

Primary Medical Indication/Disease/Condition *

e.g. "Stress", "Diabetes", or define the target group in brackets after the condition, e.g. "Autism (Parents of children with)", "Alzheimers (Informal Caregivers of)"

Not applicable — this is an educational interve

Primary Outcomes measured in trial *

comma-separated list of primary outcomes reported in the trial

Knowledge acquisition (pre/post-training chan

Secondary/other outcomes

Are there any other outcomes the intervention is expected to affect?

None beyond those listed above. The three outcome domains (knowledge, case discussion accuracy, engagement) were pre-specified as co-primary.

Recommended "Dose" *

What do the instructions for users say on how often the app should be used?

- Approximately Daily
- Approximately Weekly
- Approximately Monthly
- Approximately Yearly
- "as needed"
- (selected) Other: Single-session educational activity. Participants completed one 45–6

Approx. Percentage of Users (starters) still using the app as recommended after 3 months *

- ○ unknown / not evaluated
- ○ 0-10%
- ○ 11-20%
- ○ 21-30%
- ○ 31-40%
- ○ 41-50%
- ○ 51-60%
- ○ 61-70%
- ○ 71%-80%
- ○ 81-90%
- ○ 91-100%
- ◉ Other: NA: One time learning intervention study

Overall, was the app/intervention effective? *

- (x) yes: all primary outcomes were significantly better in intervention group vs control
- ( ) partly: SOME primary outcomes were significantly better in intervention group vs control
- ( ) no statistically significant difference between control and intervention
- ( ) potentially harmful: control was significantly better than intervention in one or more outcomes
- ( ) inconclusive: more research is needed
- ( ) Other:

Article Preparation Status/Stage *

At which stage in your article preparation are you currently (at the time you fill in this form)

- ( ) not submitted yet - in early draft status
- ( ) not submitted yet - in late draft status, just before submission
- (x) submitted to a journal but not reviewed yet
- ( ) submitted to a journal and after receiving initial reviewer comments
- ( ) submitted to a journal and accepted, but not published yet
- ( ) published
- ( ) Other:

Journal *

If you already know where you will submit this paper (or if it is already submitted), please provide the journal name (if it is not JMIR, provide the journal name under "other")

- ○ not submitted yet / unclear where I will submit this
- ○ Journal of Medical Internet Research (JMIR)
- ○ JMIR mHealth and UHealth
- ○ JMIR Serious Games
- ○ JMIR Mental Health
- ○ JMIR Public Health
- ○ JMIR Formative Research
- ○ Other JMIR sister journal
- ◉ Other: JMIR Medical Education

Is this a full powered effectiveness trial or a pilot/feasibility trial? *

- ◉ Pilot/feasibility
- ○ Fully powered

Manuscript tracking number *

If this is a JMIR submission, please provide the manuscript tracking number under "other" (The ms tracking number can be found in the submission acknowledgement email, or when you login as author in JMIR. If the paper is already published in JMIR, then the ms tracking number is the four-digit number at the end of the DOI, to be found at the bottom of each published article in JMIR)

- ○ no ms number (yet) / not (yet) submitted to / published in JMIR
- ◉ Other: 98700

TITLE AND ABSTRACT

1a) TITLE: Identification as a randomized trial in the title

1a) Does your paper address CONSORT item 1a? *

I.e does the title contain the phrase "Randomized Controlled Trial"? (if not, explain the reason under "other")

- ◉ yes
- ○ Other:

1a-i) Identify the mode of delivery in the title

Identify the mode of delivery. Preferably use “web-based” and/or “mobile” and/or “electronic game” in the title. Avoid ambiguous terms like “online”, “virtual”, “interactive”. Use “Internet-based” only if Intervention includes non-web-based Internet components (e.g. email), use “computer-based” or “electronic” only if offline products are used. Use “virtual” only in the context of “virtual reality” (3-D worlds). Use “online” only in the context of “online support groups”. Complement or substitute product names with broader terms for the class of products (such as “mobile” or “smart phone” instead of “iphone”), especially if the application runs on different platforms.

	1	2	3	4	5	
subitem not at all important	(●)	○	○	○	○	essential

Clear selection

Does your paper address subitem 1a-i? *

Copy and paste relevant sections from manuscript title (include quotes in quotation marks "like this" to indicate direct quotes from your manuscript), or elaborate on this item by providing additional information not in the ms, or briefly explain why the item is not applicable/relevant for your study

The title uses the terms “virtual patient” and “case studies,” which identify the interactive, computer-mediated mode of delivery. The intervention is an AI-enabled web-based conversational simulation as described in the abstract and methods.

1a-ii) Non-web-based components or important co-interventions in title

Mention non-web-based components or important co-interventions in title, if any (e.g., “with telephone support”).

	1	2	3	4	5	
subitem not at all important	(●)	( )	( )	( )	( )	essential

Clear selection

Does your paper address subitem 1a-ii?

Copy and paste relevant sections from manuscript title (include quotes in quotation marks "like this" to indicate direct quotes from your manuscript), or elaborate on this item by providing additional information not in the ms, or briefly explain why the item is not applicable/relevant for your study

Not applicable. There were no non-web-based components or important co-interventions other than a shared 12-minute educational video viewed by all participants before randomization, which was identical across arms and therefore not a differentiating co-intervention.

1a-iii) Primary condition or target group in the title

Mention primary condition or target group in the title, if any (e.g., “for children with Type I Diabetes”) Example: A Web-based and Mobile Intervention with Telephone Support for Children with Type I Diabetes: Randomized Controlled Trial

	1	2	3	4	5	
subitem not at all important	(●)	( )	( )	( )	( )	essential

Clear selection

Does your paper address subitem 1a-iii? *

Copy and paste relevant sections from manuscript title (include quotes in quotation marks "like this" to indicate direct quotes from your manuscript), or elaborate on this item by providing additional information not in the ms, or briefly explain why the item is not applicable/relevant for your study

Yes. The title names the target group: "…in Health Professional Students."

1b) ABSTRACT: Structured summary of trial design, methods, results, and conclusions

NPT extension: Description of experimental treatment, comparator, care providers, centers, and blinding status.

1b-i) Key features/functionalities/components of the intervention and comparator in the METHODS section of the ABSTRACT

Mention key features/functionalities/components of the intervention and comparator in the abstract. If possible, also mention theories and principles used for designing the site. Keep in mind the needs of systematic reviewers and indexers by including important synonyms. (Note: Only report in the abstract what the main paper is reporting. If this information is missing from the main body of text, consider adding it)

	1	2	3	4	5	
subitem not at all important	◉	○	○	○	○	essential

Clear selection

Does your paper address subitem 1b-i? *

Copy and paste relevant sections from the manuscript abstract (include quotes in quotation marks "like this" to indicate direct quotes from your manuscript), or elaborate on this item by providing additional information not in the ms, or briefly explain why the item is not applicable/relevant for your study

Yes. From the abstract: “Health professional students…were randomized to complete either (1) 3 static written case studies or (2) 3 interactive virtual patient case studies using the Figment Learning Labs Virtual Clinic. Case content was identical across conditions. The virtual patient system used an AI-enabled conversational interface (OpenAI GPT-4o) to deliver case material, whereas the text-based condition required reading of static case text.”

1b-ii) Level of human involvement in the METHODS section of the ABSTRACT

Clarify the level of human involvement in the abstract, e.g., use phrases like “fully automated” vs. “therapist/nurse/care provider/physician-assisted” (mention number and expertise of providers involved, if any). (Note: Only report in the abstract what the main paper is reporting. If this information is missing from the main body of text, consider adding it)

	1	2	3	4	5	
subitem not at all important	(●)	( )	( )	( )	( )	essential

Clear selection

Does your paper address subitem 1b-ii?

Copy and paste relevant sections from the manuscript abstract (include quotes in quotation marks "like this" to indicate direct quotes from your manuscript), or elaborate on this item by providing additional information not in the ms, or briefly explain why the item is not applicable/relevant for your study

Partially addressed in abstract; more fully described in Methods. The abstract states the virtual patient used “an AI-enabled conversational interface (OpenAI GPT-4o)” which implies automated delivery. The Methods section clarifies there was no human-in-the-loop during the student–virtual patient interaction: “Each comment, question, or procedure from the student is relayed to the API and the AI engine returns a response.” The feedback feature was disabled for this study; no human instructor interacted with learners during sessions.

1b-iii) Open vs. closed, web-based (self-assessment) vs. face-to-face assessments in the METHODS section of the ABSTRACT

Mention how participants were recruited (online vs. offline), e.g., from an open access website or from a clinic or a closed online user group (closed usergroup trial), and clarify if this was a purely web-based trial, or there were face-to-face components (as part of the intervention or for assessment). Clearly say if outcomes were self-assessed through questionnaires (as common in web-based trials). Note: In traditional offline trials, an open trial (open-label trial) is a type of clinical trial in which both the researchers and participants know which treatment is being administered. To avoid confusion, use “blinded” or “unblinded” to indicated the level of blinding instead of “open”, as “open” in web-based trials usually refers to “open access” (i.e. participants can self-enrol). (Note: Only report in the abstract what the main paper is reporting. If this information is missing from the main body of text, consider adding it)

	1	2	3	4	5	
subitem not at all important	◉	○	○	○	○	essential

Clear selection

Does your paper address subitem 1b-iii?

Copy and paste relevant sections from the manuscript abstract (include quotes in quotation marks "like this" to indicate direct quotes from your manuscript), or elaborate on this item by providing additional information not in the ms, or briefly explain why the item is not applicable/relevant for your study

Addressed in Methods rather than abstract. This was a closed trial (students enrolled via an announcement in a specific interprofessional course at a single US academic health sciences university). All assessments were self-administered online. From Methods: “All sessions were conducted remotely and asynchronously.” Outcomes (knowledge test, case discussion questions, engagement survey) were all self-assessed online.

1b-iv) RESULTS section in abstract must contain use data

Report number of participants enrolled/assessed in each group, the use/uptake of the intervention (e.g., attrition/adherence metrics, use over time, number of logins etc.), in addition to primary/secondary outcomes. (Note: Only report in the abstract what the main paper is reporting. If this information is missing from the main body of text, consider adding it)

	1	2	3	4	5	
subitem not at all important	◉	○	○	○	○	essential

Clear selection

Does your paper address subitem 1b-iv?

Copy and paste relevant sections from the manuscript abstract (include quotes in quotation marks "like this" to indicate direct quotes from your manuscript), or elaborate on this item by providing additional information not in the ms, or briefly explain why the item is not applicable/relevant for your study

Partially. The abstract reports enrollment-to-completion data (N=58 completers) and outcome means/statistics, but does not report per-arm attrition or per-arm use intensity. The main text provides these: “74 participants enrolled, and 58 completed all study components”; 27 (47%) in text-based group and 31 (53%) in interactive group among completers. Because the intervention was a single session, traditional metrics like logins or use-over-time are not applicable.

1b-v) CONCLUSIONS/DISCUSSION in abstract for negative trials

Conclusions/Discussions in abstract for negative trials: Discuss the primary outcome - if the trial is negative (primary outcome not changed), and the intervention was not used, discuss whether negative results are attributable to lack of uptake and discuss reasons. (Note: Only report in the abstract what the main paper is reporting. If this information is missing from the main body of text, consider adding it)

	1	2	3	4	5	
subitem not at all important	(●)	○	○	○	○	essential

Clear selection

Does your paper address subitem 1b-v?

Copy and paste relevant sections from the manuscript abstract (include quotes in quotation marks "like this" to indicate direct quotes from your manuscript), or elaborate on this item by providing additional information not in the ms, or briefly explain why the item is not applicable/relevant for your study

Not applicable. The trial was positive across all three primary outcome domains (knowledge, case discussion accuracy, engagement).

INTRODUCTION

2a) In INTRODUCTION: Scientific background and explanation of rationale

2a-i) Problem and the type of system/solution

Describe the problem and the type of system/solution that is object of the study: intended as stand-alone intervention vs. incorporated in broader health care program? Intended for a particular patient population? Goals of the intervention, e.g., being more cost-effective to other interventions, replace or complement other solutions? (Note: Details about the intervention are provided in "Methods" under 5)

	1	2	3	4	5	
subitem not at all important	(●)	( )	( )	( )	( )	essential

Clear selection

Does your paper address subitem 2a-i? *

Copy and paste relevant sections from the manuscript (include quotes in quotation marks "like this" to indicate direct quotes from your manuscript), or elaborate on this item by providing additional information not in the ms, or briefly explain why the item is not applicable/relevant for your study

Yes. From the Introduction: “Effective pain management education, particularly regarding opioid risks, remains a challenge in health professions training.” “Traditional approaches, including lectures, readings, and static case studies, often fall short of building the clinical reasoning skills required to address the complexities of patient-centered pain management.” The Figment Clinic is presented as a scalable, interactive alternative intended to complement (not replace) existing simulation-based learning: “Advances in digital simulation technologies and conversational interfaces have expanded opportunities for scalable, interactive learning environments…”

2a-ii) Scientific background, rationale: What is known about the (type of) system

Scientific background, rationale: What is known about the (type of) system that is the object of the study (be sure to discuss the use of similar systems for other conditions/diagnoses, if appropiate), motivation for the study, i.e. what are the reasons for and what is the context for this specific study, from which stakeholder viewpoint is the study performed, potential impact of findings [2]. Briefly justify the choice of the comparator.

	1	2	3	4	5	
subitem not at all important	◉	○	○	○	○	essential

Clear selection

Does your paper address subitem 2a-ii? *

Copy and paste relevant sections from the manuscript (include quotes in quotation marks "like this" to indicate direct quotes from your manuscript), or elaborate on this item by providing additional information not in the ms, or briefly explain why the item is not applicable/relevant for your study

Yes. The Introduction reviews: (a) evidence that simulation-based learning improves outcomes but is resource-intensive (references 5–11); (b) emerging evidence on virtual patients and natural-language interfaces (reference 12); (c) active vs. passive learning as a confound in this literature (references 13–15); and (d) prior LLM-driven virtual patient work (references 16–18). The comparator (written case studies) is explicitly justified: "Static case studies and lecture-based instruction are widely used but may be insufficient to develop the clinical reasoning and communication skills required in practice."

2b) In INTRODUCTION: Specific objectives or hypotheses

Does your paper address CONSORT subitem 2b? *

Copy and paste relevant sections from the manuscript (include quotes in quotation marks "like this" to indicate direct quotes from your manuscript), or elaborate on this item by providing additional information not in the ms, or briefly explain why the item is not applicable/relevant for your study

Yes. From the Objective section: "Within this context, the present randomized study sought to examine whether learning outcomes differed when educational cases were delivered through interactive virtual patient encounters compared with static written case studies. Specifically, the study evaluated differences in knowledge acquisition, case discussion quiz performance, and learner engagement among health professional students learning about pain and opioid management."

METHODS

3a) Description of trial design (such as parallel, factorial) including allocation ratio

Does your paper address CONSORT subitem 3a? *

Copy and paste relevant sections from the manuscript (include quotes in quotation marks "like this" to indicate direct quotes from your manuscript), or elaborate on this item by providing additional information not in the ms, or briefly explain why the item is not applicable/relevant for your study

Yes. The design is a two-arm parallel-group randomized controlled trial with 1:1 allocation. From Methods: “Participants were randomized 1:1 by the software platform into 1 of 2 groups.” The abstract also describes it as a randomized controlled trial. All participants watched the same 12-minute video and completed identical pre/post assessments; the only manipulated factor was case delivery format.

3b) Important changes to methods after trial commencement (such as eligibility criteria), with reasons

Does your paper address CONSORT subitem 3b? *

Copy and paste relevant sections from the manuscript (include quotes in quotation marks "like this" to indicate direct quotes from your manuscript), or elaborate on this item by providing additional information not in the ms, or briefly explain why the item is not applicable/relevant for your study

No changes were made to eligibility criteria, outcomes, or randomization procedures after the trial commenced.

3b-i) Bug fixes, Downtimes, Content Changes

Bug fixes, Downtimes, Content Changes: ehealth systems are often dynamic systems. A description of changes to methods therefore also includes important changes made on the intervention or comparator during the trial (e.g., major bug fixes or changes in the functionality or content) (5-iii) and other “unexpected events” that may have influenced study design such as staff changes, system failures/downtimes, etc. [2].

	1	2	3	4	5	
subitem not at all important	(●)	( )	( )	( )	( )	essential

Clear selection

Does your paper address subitem 3b-i?

Copy and paste relevant sections from the manuscript (include quotes in quotation marks "like this" to indicate direct quotes from your manuscript), or elaborate on this item by providing additional information not in the ms, or briefly explain why the item is not applicable/relevant for your study

No material changes, bug fixes, or downtime affected the study. Figment Clinic version 1.0 was used throughout, and content was frozen at the start of enrollment on April 1, 2025. No unexpected events (staff changes, platform outages) occurred during the study period.

4a) Eligibility criteria for participants

Does your paper address CONSORT subitem 4a? *

Copy and paste relevant sections from the manuscript (include quotes in quotation marks "like this" to indicate direct quotes from your manuscript), or elaborate on this item by providing additional information not in the ms, or briefly explain why the item is not applicable/relevant for your study

Yes. From Methods: “Participants were health professional students enrolled at a US-based academic health sciences university, representing programs in medicine, dentistry, nursing, pharmacy, and allied health. Inclusion criteria required active enrollment in clinical or pre-clinical coursework. An announcement about the availability of this study opportunity was made during an interprofessional course in the spring semester. Participation was voluntary.”

4a-i) Computer / Internet literacy

Computer / Internet literacy is often an implicit “de facto” eligibility criterion - this should be explicitly clarified.

	1	2	3	4	5	
subitem not at all important	●	○	○	○	○	essential

Clear selection

Does your paper address subitem 4a-i?

Copy and paste relevant sections from the manuscript (include quotes in quotation marks "like this" to indicate direct quotes from your manuscript), or elaborate on this item by providing additional information not in the ms, or briefly explain why the item is not applicable/relevant for your study

Not explicitly stated as an inclusion criterion, but implicit. All participants were currently enrolled health professional students at a US academic health sciences university, where routine coursework and assessment are delivered through learning management systems. Digital literacy sufficient to access a website, watch a video, and complete online questionnaires was effectively required.

4a-ii) Open vs. closed, web-based vs. face-to-face assessments:

Open vs. closed, web-based vs. face-to-face assessments: Mention how participants were recruited (online vs. offline), e.g., from an open access website or from a clinic, and clarify if this was a purely web-based trial, or there were face-to-face components (as part of the intervention or for assessment), i.e., to what degree got the study team to know the participant. In online-only trials, clarify if participants were quasi-anonymous and whether having multiple identities was possible or whether technical or logistical measures (e.g., cookies, email confirmation, phone calls) were used to detect/prevent these.

	1	2	3	4	5	
subitem not at all important	(•)	( )	( )	( )	( )	essential

Clear selection

Does your paper address subitem 4a-ii? *

Copy and paste relevant sections from the manuscript (include quotes in quotation marks "like this" to indicate direct quotes from your manuscript), or elaborate on this item by providing additional information not in the ms, or briefly explain why the item is not applicable/relevant for your study

This was a closed trial. Recruitment was via an in-person announcement during an interprofessional course at one US academic health sciences university. Students self-enrolled by visiting the study website, reviewing the informed consent waiver, and completing a demographics form. The identity of participants was known at the institutional level (students were enrolled at the single site) but individual identifying information was not shared with the AI model: “No participant identifying information was shared with the AI model or retained by the AI provider.” Multiple identities were not possible because enrollment was tied to institutionally-known students via the course. All assessments (knowledge test, discussion questions, engagement survey) were web-based and self-administered. There were no face-to-face components.

4a-iii) Information giving during recruitment

Information given during recruitment. Specify how participants were briefed for recruitment and in the informed consent procedures (e.g., publish the informed consent documentation as appendix, see also item X26), as this information may have an effect on user self-selection, user expectation and may also bias results.

	1	2	3	4	5	
subitem not at all important	(●)	( )	( )	( )	( )	essential

Clear selection

Does your paper address subitem 4a-iii?

Copy and paste relevant sections from the manuscript (include quotes in quotation marks "like this" to indicate direct quotes from your manuscript), or elaborate on this item by providing additional information not in the ms, or briefly explain why the item is not applicable/relevant for your study

From Methods: “Participants visited the study website where they reviewed the informed consent waiver online, completed a demographics form, and completed a baseline knowledge assessment, and were then randomized.” The announcement during the interprofessional course described the study opportunity; the online informed consent waiver was the primary recruitment information document.

4b) Settings and locations where the data were collected

Does your paper address CONSORT subitem 4b? *

Copy and paste relevant sections from the manuscript (include quotes in quotation marks "like this" to indicate direct quotes from your manuscript), or elaborate on this item by providing additional information not in the ms, or briefly explain why the item is not applicable/relevant for your study

Yes. From Methods: “All sessions were conducted remotely and asynchronously, and lasted approximately 45–60 minutes.” Participants accessed the study website from their own devices, at a location of their choosing. All data were collected from a single US academic health sciences university.

4b-i) Report if outcomes were (self-)assessed through online questionnaires

Clearly report if outcomes were (self-)assessed through online questionnaires (as common in web-based trials) or otherwise.

	1	2	3	4	5	
subitem not at all important	◉	○	○	○	○	essential

Clear selection

Does your paper address subitem 4b-i? *

Copy and paste relevant sections from the manuscript (include quotes in quotation marks "like this" to indicate direct quotes from your manuscript), or elaborate on this item by providing additional information not in the ms, or briefly explain why the item is not applicable/relevant for your study

Yes, explicitly. All outcomes were self-assessed online: the 8-item knowledge test (pre and post), the 9-item case discussion quiz (administered after each of three cases), and the 17-item learner engagement survey were all delivered online through the study platform.

4b-ii) Report how institutional affiliations are displayed

Report how institutional affiliations are displayed to potential participants [on ehealth media], as affiliations with prestigious hospitals or universities may affect volunteer rates, use, and reactions with regards to an intervention.(Not a required item – describe only if this may bias results)

	1	2	3	4	5	
subitem not at all important	(•)	( )	( )	( )	( )	essential

Clear selection

Does your paper address subitem 4b-ii?

Copy and paste relevant sections from the manuscript (include quotes in quotation marks "like this" to indicate direct quotes from your manuscript), or elaborate on this item by providing additional information not in the ms, or briefly explain why the item is not applicable/relevant for your study

Not applicable to outcomes. The study was conducted among students already enrolled at MUSC, and the platform affiliation was not novel information to participants.

5) The interventions for each group with sufficient details to allow replication, including how and when they were actually administered

5-i) Mention names, credential, affiliations of the developers, sponsors, and owners

Mention names, credential, affiliations of the developers, sponsors, and owners [6] (if authors/evaluators are owners or developer of the software, this needs to be declared in a “Conflict of interest” section or mentioned elsewhere in the manuscript).

	1	2	3	4	5	
subitem not at all important	(●)	( )	( )	( )	( )	essential

Clear selection

Does your paper address subitem 5-i?

Copy and paste relevant sections from the manuscript (include quotes in quotation marks "like this" to indicate direct quotes from your manuscript), or elaborate on this item by providing additional information not in the ms, or briefly explain why the item is not applicable/relevant for your study

Yes. From the AI Model Information section: “For this study, Figment Clinic version 1.0 (Palmetto Innovative Education LLC, doing business as Figment Learning Labs; Mount Pleasant, SC) was used.” The Conflicts of Interest section discloses: “JJB and DH are employed by the Medical University of South Carolina (MUSC) and are founders of Palmetto Innovative Education LLC, doing business as Figment Learning Labs — the company formed to commercialize the Figment Learning Labs software developed at MUSC through NIH funding.” Funding: “This work was funded by National Institutes of Health (NIH) Small Business Technology Transfer (STTR) Grant 1R41DA059281-01A1.”

5-ii) Describe the history/development process

Describe the history/development process of the application and previous formative evaluations (e.g., focus groups, usability testing), as these will have an impact on adoption/use rates and help with interpreting results.

	1	2	3	4	5	
subitem not at all important	◉	○	○	○	○	essential

Clear selection

Does your paper address subitem 5-ii?

Copy and paste relevant sections from the manuscript (include quotes in quotation marks "like this" to indicate direct quotes from your manuscript), or elaborate on this item by providing additional information not in the ms, or briefly explain why the item is not applicable/relevant for your study

The platform was developed at MUSC through NIH STTR funding (Grant 1R41DA059281-01A1). Formative usability testing was conducted during development by members of the investigator team and a small sample of faculty and students, though formal focus-group findings are not reported in this manuscript. The three cases used in the study were authored and reviewed by the investigative team for content validity prior to the trial.

5-iii) Revisions and updating

Revisions and updating. Clearly mention the date and/or version number of the application/intervention (and comparator, if applicable) evaluated, or describe whether the intervention underwent major changes during the evaluation process, or whether the development and/or content was “frozen” during the trial. Describe dynamic components such as news feeds or changing content which may have an impact on the replicability of the intervention (for unexpected events see item 3b).

	1	2	3	4	5	
subitem not at all important	◉	○	○	○	○	essential

Clear selection

Does your paper address subitem 5-iii?

Copy and paste relevant sections from the manuscript (include quotes in quotation marks "like this" to indicate direct quotes from your manuscript), or elaborate on this item by providing additional information not in the ms, or briefly explain why the item is not applicable/relevant for your study

Figment Clinic version 1.0 was used throughout the trial. Case content was frozen at the start of enrollment on April 1, 2025 and did not change during the data collection period ending July 31, 2025. No major version updates or content changes occurred during the study.

5-iv) Quality assurance methods

Provide information on quality assurance methods to ensure accuracy and quality of information provided [1], if applicable.

	1	2	3	4	5	
subitem not at all important	(●)	○	○	○	○	essential

Clear selection

Does your paper address subitem 5-iv?

Copy and paste relevant sections from the manuscript (include quotes in quotation marks "like this" to indicate direct quotes from your manuscript), or elaborate on this item by providing additional information not in the ms, or briefly explain why the item is not applicable/relevant for your study

The three case studies were developed by the investigative team and reviewed for content accuracy and alignment with the learning objectives of the 12-minute training video. Correct answers to the three case discussion quiz questions were determined a priori by the investigators. From Methods: “Question stems were developed and correct answers were determined by consensus of the investigative team; interrater reliability among authors was not formally assessed.” Within the platform, author-specified system prompts and guardrails were used to constrain the AI model from deviating from the virtual patient role.

5-v) Ensure replicability by publishing the source code, and/or providing screenshots/screen-capture video, and/or providing flowcharts of the algorithms used

Ensure replicability by publishing the source code, and/or providing screenshots/screen-capture video, and/or providing flowcharts of the algorithms used. Replicability (i.e., other researchers should in principle be able to replicate the study) is a hallmark of scientific reporting.

	1	2	3	4	5	
subitem not at all important	○	○	○	○	○	essential

Does your paper address subitem 5-v?

Copy and paste relevant sections from the manuscript (include quotes in quotation marks "like this" to indicate direct quotes from your manuscript), or elaborate on this item by providing additional information not in the ms, or briefly explain why the item is not applicable/relevant for your study

Figure 1 provides screenshots of both conditions: Figure 1A shows the static text-based case presentation format and Figure 1B shows the Figment Clinic virtual patient interface. The Methods section describes the platform architecture, OpenAI API integration, case-authoring fields (demographics, history, procedures, AI behavior instructions), and the full set of model parameters (temperature=0.7, top_p=0.9, frequency_penalty=0.8, presence_penalty=0.8, max_tokens=500). Case content is summarized in Multimedia Appendix 2. Source code is not publicly released because the platform is proprietary commercial software, but the case-authoring parameters, model parameters, and case content are fully described in the manuscript and appendices.

5-vi) Digital preservation

Digital preservation: Provide the URL of the application, but as the intervention is likely to change or disappear over the course of the years; also make sure the intervention is archived (Internet Archive, webcitation.org, and/or publishing the source code or screenshots/videos alongside the article). As pages behind login screens cannot be archived, consider creating demo pages which are accessible without login.

	1	2	3	4	5	
subitem not at all important	(selected)	○	○	○	○	essential

Clear selection

Does your paper address subitem 5-vi?

Copy and paste relevant sections from the manuscript (include quotes in quotation marks "like this" to indicate direct quotes from your manuscript), or elaborate on this item by providing additional information not in the ms, or briefly explain why the item is not applicable/relevant for your study

The Figment Clinic platform is maintained at https://figmenthealth.com by Palmetto Innovative Education LLC. The case-authoring parameters and case content used in this study are preserved in Multimedia Appendix 2. Screenshots of both conditions are preserved in Figure 1. Because case materials behind a login cannot be publicly archived, the Multimedia Appendices serve as the permanent public record of the intervention content.

5-vii) Access

Access: Describe how participants accessed the application, in what setting/context, if they had to pay (or were paid) or not, whether they had to be a member of specific group. If known, describe how participants obtained “access to the platform and Internet” [1]. To ensure access for editors/reviewers/readers, consider to provide a “backdoor” login account or demo mode for reviewers/readers to explore the application (also important for archiving purposes, see vi).

	1	2	3	4	5	
subitem not at all important	(●)	( )	( )	( )	( )	essential

Clear selection

Does your paper address subitem 5-vii? *

Copy and paste relevant sections from the manuscript (include quotes in quotation marks "like this" to indicate direct quotes from your manuscript), or elaborate on this item by providing additional information not in the ms, or briefly explain why the item is not applicable/relevant for your study

From Methods: “Study enrollment opened on April 1, 2025, and closed on July 31, 2025. Participants visited the study website where they reviewed the informed consent waiver online.” Participants accessed the platform without payment. Access was restricted to students enrolled in the specific interprofessional course who responded to the in-person announcement.

5-viii) Mode of delivery, features/functionalities/components of the intervention and comparator, and the theoretical framework

Describe mode of delivery, features/functionalities/components of the intervention and comparator, and the theoretical framework [6] used to design them (instructional strategy [1], behaviour change techniques, persuasive features, etc., see e.g., [7, 8] for terminology). This includes an in-depth description of the content (including where it is coming from and who developed it) [1],” whether [and how] it is tailored to individual circumstances and allows users to track their progress and receive feedback” [6]. This also includes a description of communication delivery channels and – if computer-mediated communication is a component – whether communication was synchronous or asynchronous [6]. It also includes information on presentation strategies [1], including page design principles, average amount of text on pages, presence of hyperlinks to other resources, etc. [1].

	1	2	3	4	5	
subitem not at all important	◉	○	○	○	○	essential

Clear selection

Does your paper address subitem 5-viii? *

Copy and paste relevant sections from the manuscript (include quotes in quotation marks "like this" to indicate direct quotes from your manuscript), or elaborate on this item by providing additional information not in the ms, or briefly explain why the item is not applicable/relevant for your study

Yes, extensively. From the AI-Powered Virtual Patients section: “The Figment Learning Labs Virtual Clinic is an online system that permits course facilitators to develop AI-powered interactive case studies that students can interview. Virtual patient avatars (selectable by the facilitator) are animated and superimposed on a cartoon clinic backdrop (also selectable). The interface includes a medical record for each patient that can be viewed during the interview, an editable visit/progress note, and a full transcript history available afterwards for review.” “Instructors specify learning objectives for each case along with key patient information: name, age, sex, race, ethnicity, height, weight, personality, mood during the interview, wordiness, chief complaint, history of present illness, social history, family and medical history, medication list, allergies, and other relevant medical information.” “At the beginning of a clinic session, virtual patient background information is sent…as a system-level prompt, along with the general context and rationale for the activity and guardrail instructions to prevent the AI from deviating from the role of virtual patient.” “The format is open: learners can guide the interview in any direction they deem clinically fit.” “Interviews are online, asynchronous, and interactive, and can be conducted via voice or text/typing interfaces.” The comparator was static, formatted written case vignettes with an accompanying patient photograph. Theoretical framework: the intervention is grounded in simulation-based learning principles (deliberate practice, cognitive framing, fidelity alignment) and in the active-learning literature, both of which are discussed in the Introduction.

5-ix) Describe use parameters

Describe use parameters (e.g., intended “doses” and optimal timing for use). Clarify what instructions or recommendations were given to the user, e.g., regarding timing, frequency, heaviness of use, if any, or was the intervention used ad libitum.

	1	2	3	4	5	
subitem not at all important	(x)	( )	( )	( )	( )	essential

Clear selection

Does your paper address subitem 5-ix?

Copy and paste relevant sections from the manuscript (include quotes in quotation marks "like this" to indicate direct quotes from your manuscript), or elaborate on this item by providing additional information not in the ms, or briefly explain why the item is not applicable/relevant for your study

Single-session protocol. All participants completed 3 cases in one session. No daily/weekly “dose” instructions applied. Participants in the interactive arm were not given a time limit for interviews but were informed each session would take approximately 45–60 minutes total (video + cases + assessments). Participants were not permitted to return to case material after starting the discussion quiz.

5-x) Clarify the level of human involvement

Clarify the level of human involvement (care providers or health professionals, also technical assistance) in the e-intervention or as co-intervention (detail number and expertise of professionals involved, if any, as well as “type of assistance offered, the timing and frequency of the support, how it is initiated, and the medium by which the assistance is delivered”. It may be necessary to distinguish between the level of human involvement required for the trial, and the level of human involvement required for a routine application outside of a RCT setting (discuss under item 21 – generalizability).

	1	2	3	4	5	
subitem not at all important	(●)	( )	( )	( )	( )	essential

Clear selection

Does your paper address subitem 5-x?

Copy and paste relevant sections from the manuscript (include quotes in quotation marks "like this" to indicate direct quotes from your manuscript), or elaborate on this item by providing additional information not in the ms, or briefly explain why the item is not applicable/relevant for your study

Fully automated student–virtual patient interaction during the case sessions. No human instructor or study staff member interacted with learners during the intervention. From Methods: “While learner feedback is an optional feature of the Figment virtual clinic platform, the feedback feature was disabled for this study; no learners received feedback about their interview, discussion question, or knowledge test performance.” Technical support (should a participant have needed it) was available by email to the study team but was not used during the study.

5-xi) Report any prompts/reminders used

Report any prompts/reminders used: Clarify if there were prompts (letters, emails, phone calls, SMS) to use the application, what triggered them, frequency etc. It may be necessary to distinguish between the level of prompts/reminders required for the trial, and the level of prompts/reminders for a routine application outside of a RCT setting (discuss under item 21 – generalizability).

	1	2	3	4	5	
subitem not at all important	◉	○	○	○	○	essential

Clear selection

Does your paper address subitem 5-xi? *

Copy and paste relevant sections from the manuscript (include quotes in quotation marks "like this" to indicate direct quotes from your manuscript), or elaborate on this item by providing additional information not in the ms, or briefly explain why the item is not applicable/relevant for your study

Not applicable. Because the study was a single session completed in one sitting, no prompts or reminders to return to the intervention were used. Participants who began the study and did not complete it (n=16) were not contacted to re-engage.

5-xii) Describe any co-interventions (incl. training/support)

Describe any co-interventions (incl. training/support): Clearly state any interventions that are provided in addition to the targeted eHealth intervention, as ehealth intervention may not be designed as stand-alone intervention. This includes training sessions and support [1]. It may be necessary to distinguish between the level of training required for the trial, and the level of training for a routine application outside of a RCT setting (discuss under item 21 – generalizability.

	1	2	3	4	5	
subitem not at all important	(●)	( )	( )	( )	( )	essential

Clear selection

Does your paper address subitem 5-xii? *

Copy and paste relevant sections from the manuscript (include quotes in quotation marks "like this" to indicate direct quotes from your manuscript), or elaborate on this item by providing additional information not in the ms, or briefly explain why the item is not applicable/relevant for your study

The only co-intervention was the shared 12-minute educational video on evidence-based pain and opioid management, which was viewed by all participants in both arms before case assignment. From Methods: “All participants were exposed to the same didactic training video, and participants were unable to skip video content during the study.” No platform-orientation training was provided because the interface is designed to be self-explanatory; no usability issues were reported during the study. No technical support was delivered during the study.

6a) Completely defined pre-specified primary and secondary outcome measures, including how and when they were assessed

Does your paper address CONSORT subitem 6a? *

Copy and paste relevant sections from the manuscript (include quotes in quotation marks "like this" to indicate direct quotes from your manuscript), or elaborate on this item by providing additional information not in the ms, or briefly explain why the item is not applicable/relevant for your study

Yes. Three pre-specified outcome domains are defined in Methods: (1) Knowledge Assessment - “a brief, custom 8-item multiple-choice test…administered pre- and post-training”; (2) Case Discussion Quiz Accuracy - “3 multiple-choice discussion questions” administered after each of 3 cases, scored 1 point per correct answer; (3) Learner Engagement Survey - “17 items designed to assess online interface usability and value, learner engagement and satisfaction with the learning experience, and applicability of the information learned to real-world scenarios” on a 5-point Likert scale. Each instrument’s full item set is provided in Multimedia Appendices 1 and 3.

6a-i) Online questionnaires: describe if they were validated for online use and apply CHERRIES items to describe how the questionnaires were designed/deployed

If outcomes were obtained through online questionnaires, describe if they were validated for online use and apply CHERRIES items to describe how the questionnaires were designed/deployed [9].

	1	2	3	4	5	
subitem not at all important	(●)	( )	( )	( )	( )	essential

Clear selection

Does your paper address subitem 6a-i?

Copy and paste relevant sections from manuscript text

The three study instruments (8-item knowledge test, 9-item case discussion quiz, 17-item engagement survey) were developed by the investigative team for this trial and have not been formally validated outside of this study. This limitation is acknowledged in the manuscript: “the assessment instruments were brief, limiting the granularity with which learning gains could be measured…future studies should consider incorporating validated multifaceted assessments.” Internal consistency was assessed: Cronbach α=.56 (knowledge test), .31 (case discussion; limitation discussed as reflecting heterogeneous cases), and .95 (engagement survey). All questionnaires were deployed as standard web forms with no adaptive or skip logic; items were presented in fixed order; no completeness checks beyond standard form validation were used.

6a-ii) Describe whether and how “use” (including intensity of use/dosage) was defined/measured/monitored

Describe whether and how “use” (including intensity of use/dosage) was defined/measured/monitored (logins, logfile analysis, etc.). Use/adoption metrics are important process outcomes that should be reported in any ehealth trial.

	1	2	3	4	5	
subitem not at all important	(●)	( )	( )	( )	( )	essential

Clear selection

Does your paper address subitem 6a-ii?

Copy and paste relevant sections from manuscript text

Use was defined as completion of all study components (video, 3 cases, pre/post knowledge test, engagement survey). Of 74 participants who enrolled, 58 (78%) completed all components and were analyzed. Per-arm time-on-task was not recorded; this is acknowledged as a limitation: “time-on-task was not measured. Students in the virtual patient group may have spent more time interacting with the virtual patient cases than learners assigned to the static vignettes…we cannot disentangle the influence of duration of engagement from the intrinsic effects of the simulation modality.” Platform-level log data were not analyzed for this report.

6a-iii) Describe whether, how, and when qualitative feedback from participants was obtained

Describe whether, how, and when qualitative feedback from participants was obtained (e.g., through emails, feedback forms, interviews, focus groups).

	1	2	3	4	5	
subitem not at all important	○	○	○	○	○	essential

Does your paper address subitem 6a-iii?

Copy and paste relevant sections from manuscript text

Qualitative free-text responses were not systematically collected as part of this study. The 17-item engagement survey (quantitative Likert items) was the sole source of learner feedback on the experience.

6b) Any changes to trial outcomes after the trial commenced, with reasons

Does your paper address CONSORT subitem 6b? *

Copy and paste relevant sections from the manuscript (include quotes in quotation marks "like this" to indicate direct quotes from your manuscript), or elaborate on this item by providing additional information not in the ms, or briefly explain why the item is not applicable/relevant for your study

No changes were made to the outcomes after the trial commenced. The three pre-specified outcome domains (knowledge, case discussion accuracy, engagement) were analyzed exactly as planned.

7a) How sample size was determined

NPT: When applicable, details of whether and how the clustering by care provides or centers was addressed

7a-i) Describe whether and how expected attrition was taken into account when calculating the sample size

Describe whether and how expected attrition was taken into account when calculating the sample size.

	1	2	3	4	5	
subitem not at all important	◉	○	○	○	○	essential

Clear selection

Does your paper address subitem 7a-i?

Copy and paste relevant sections from manuscript title (include quotes in quotation marks "like this" to indicate direct quotes from your manuscript), or elaborate on this item by providing additional information not in the ms, or briefly explain why the item is not applicable/relevant for your study

The manuscript does not report a formal a priori power calculation. Recruitment was by voluntary enrollment from a single course during one semester, which capped the available pool. This study is described in the manuscript as “preliminary in nature and primarily hypothesis generating” and is framed as a pilot/feasibility-stage RCT; larger fully-powered multi-institutional trials are identified as a future direction. The final sample of 58 completers was the full available pool from the enrolling course. No formal a priori sample-size calculation was performed; see 7a above. Expected attrition was not explicitly factored in at the design stage. Observed attrition (16/74 = 21.6%) is reported and analyzed in the Attrition section of Results.

7b) When applicable, explanation of any interim analyses and stopping guidelines

Does your paper address CONSORT subitem 7b? *

Copy and paste relevant sections from the manuscript (include quotes in quotation marks "like this" to indicate direct quotes from your manuscript), or elaborate on this item by providing additional information not in the ms, or briefly explain why the item is not applicable/relevant for your study

Not applicable. No interim analyses were conducted and no stopping rules were pre-specified. The trial ran to the planned end-of-recruitment date (July 31, 2025).

8a) Method used to generate the random allocation sequence

NPT: When applicable, how care providers were allocated to each trial group

Does your paper address CONSORT subitem 8a? *

Copy and paste relevant sections from the manuscript (include quotes in quotation marks "like this" to indicate direct quotes from your manuscript), or elaborate on this item by providing additional information not in the ms, or briefly explain why the item is not applicable/relevant for your study

From Methods: “Participants were randomized 1:1 by the software platform into 1 of 2 groups.” Allocation was generated programmatically by the study web application at the point of each participant’s enrollment.

8b) Type of randomisation; details of any restriction (such as blocking and block size)

Does your paper address CONSORT subitem 8b? *

Copy and paste relevant sections from the manuscript (include quotes in quotation marks "like this" to indicate direct quotes from your manuscript), or elaborate on this item by providing additional information not in the ms, or briefly explain why the item is not applicable/relevant for your study

Simple 1:1 randomization by software. No block randomization, stratification, or restriction was used. The manuscript does not report the specific randomization algorithm (e.g., simple vs. block); investigators can confirm this detail at revision if requested.

9) Mechanism used to implement the random allocation sequence (such as sequentially numbered containers), describing any steps taken to conceal the sequence until interventions were assigned

Does your paper address CONSORT subitem 9? *

Copy and paste relevant sections from the manuscript (include quotes in quotation marks "like this" to indicate direct quotes from your manuscript), or elaborate on this item by providing additional information not in the ms, or briefly explain why the item is not applicable/relevant for your study

Allocation was performed by the study web application at the point of enrollment, immediately after completion of the online informed consent waiver, demographics form, and baseline knowledge assessment. Because the platform assigned groups automatically and participants could not view or alter their allocation, allocation concealment was inherent to the system design. Investigators did not interact with participants at the point of allocation and could not influence group assignment.

10) Who generated the random allocation sequence, who enrolled participants, and who assigned participants to interventions

Does your paper address CONSORT subitem 10? *

Copy and paste relevant sections from the manuscript (include quotes in quotation marks "like this" to indicate direct quotes from your manuscript), or elaborate on this item by providing additional information not in the ms, or briefly explain why the item is not applicable/relevant for your study

The random allocation was generated and implemented by the study web application. Participants self-enrolled through the study website after viewing the in-course announcement. No member of the investigative team personally allocated participants to arms.

11a) If done, who was blinded after assignment to interventions (for example, participants, care providers, those assessing outcomes) and how

NPT: Whether or not administering co-interventions were blinded to group assignment

11a-i) Specify who was blinded, and who wasn't

Specify who was blinded, and who wasn't. Usually, in web-based trials it is not possible to blind the participants [1, 3] (this should be clearly acknowledged), but it may be possible to blind outcome assessors, those doing data analysis or those administering co-interventions (if any).

	1	2	3	4	5	
subitem not at all important	(●)	( )	( )	( )	( )	essential

Clear selection

Does your paper address subitem 11a-i? *

Copy and paste relevant sections from the manuscript (include quotes in quotation marks "like this" to indicate direct quotes from your manuscript), or elaborate on this item by providing additional information not in the ms, or briefly explain why the item is not applicable/relevant for your study

Participant blinding was not possible because the two conditions (reading written cases vs. interviewing an AI-driven virtual patient) are visibly distinct. Investigators involved in subsequent statistical analysis were aware of group assignment. Outcome assessment was conducted automatically through the online platform (not by a human rater), so assessor blinding was not applicable.

11a-ii) Discuss e.g., whether participants knew which intervention was the "intervention of interest" and which one was the "comparator"

Informed consent procedures (4a-ii) can create biases and certain expectations - discuss e.g., whether participants knew which intervention was the "intervention of interest" and which one was the "comparator".

	1	2	3	4	5	
subitem not at all important	○	○	○	○	○	essential

Does your paper address subitem 11a-ii?

Copy and paste relevant sections from the manuscript (include quotes in quotation marks "like this" to indicate direct quotes from your manuscript), or elaborate on this item by providing additional information not in the ms, or briefly explain why the item is not applicable/relevant for your study

The online informed consent document described the study as comparing two educational formats (written vs. interactive virtual patient) and did not signal which arm the investigators considered the "intervention of interest." However, it is plausible that some participants inferred the interactive format to be the novel condition. This expectancy effect is a known confound in educational intervention research and is discussed as part of the broader limitation that "the effects of AI cannot be separated from the broader benefits of active learning."

11b) If relevant, description of the similarity of interventions

(this item is usually not relevant for ehealth trials as it refers to similarity of a placebo or sham intervention to a active medication/intervention)

Does your paper address CONSORT subitem 11b? *

Copy and paste relevant sections from the manuscript (include quotes in quotation marks "like this" to indicate direct quotes from your manuscript), or elaborate on this item by providing additional information not in the ms, or briefly explain why the item is not applicable/relevant for your study

Not applicable. This item addresses placebo/sham similarity in pharmacological trials. The two conditions in this trial are intentionally dissimilar in mode of delivery (static vs. interactive), which is the experimental manipulation of interest.

12a) Statistical methods used to compare groups for primary and secondary outcomes

NPT: When applicable, details of whether and how the clustering by care providers or centers was addressed

Does your paper address CONSORT subitem 12a? *

Copy and paste relevant sections from the manuscript (include quotes in quotation marks "like this" to indicate direct quotes from your manuscript), or elaborate on this item by providing additional information not in the ms, or briefly explain why the item is not applicable/relevant for your study

Yes. From Statistical Analysis: “Knowledge gains were assessed via 2×2 mixed ANOVA, examining scores (percent correct) on the knowledge test pre- and post-training and between groups (static cases vs interactive cases). Case discussion question accuracy and learner self-report satisfaction and perceptions were compared between the 2 case conditions using independent-samples t tests. Analyses were conducted in SPSS version 27 (IBM Corp), with the critical alpha set at .05.”

12a-i) Imputation techniques to deal with attrition / missing values

Imputation techniques to deal with attrition / missing values: Not all participants will use the intervention/comparator as intended and attrition is typically high in ehealth trials. Specify how participants who did not use the application or dropped out from the trial were treated in the statistical analysis (a complete case analysis is strongly discouraged, and simple imputation techniques such as LOCF may also be problematic [4]).

	1	2	3	4	5	
subitem not at all important	◉	○	○	○	○	essential

Clear selection

Does your paper address subitem 12a-i? *

Copy and paste relevant sections from the manuscript (include quotes in quotation marks "like this" to indicate direct quotes from your manuscript), or elaborate on this item by providing additional information not in the ms, or briefly explain why the item is not applicable/relevant for your study

The analysis was a complete-case analysis. The 16 participants who discontinued before the case study portion were dropped from analysis after confirming no systematic differences in demographics between completers and non-completers. From Methods: “After we confirmed that study discontinuation was not associated with differences in sex, race, ethnicity, degree program, or age…the 16 non-completers were dropped from the analysis, which was then conducted only on the 58 participants who completed the study.” No imputation was performed. This is a limitation, acknowledged in the manuscript, and more sophisticated approaches (e.g., ITT with multiple imputation) were not used given all 16 non-completers dropped out before the case portion (i.e., before any outcome data were available to impute).

12b) Methods for additional analyses, such as subgroup analyses and adjusted analyses

Does your paper address CONSORT subitem 12b? *

Copy and paste relevant sections from the manuscript (include quotes in quotation marks "like this" to indicate direct quotes from your manuscript), or elaborate on this item by providing additional information not in the ms, or briefly explain why the item is not applicable/relevant for your study

No pre-specified subgroup analyses or covariate adjustments were performed. Baseline demographic and pre-test-score comparisons (Table 1) were reported as balance checks, not as inferential subgroup analyses. No exploratory subgroup analyses are reported.

X26) REB/IRB Approval and Ethical Considerations [recommended as subheading under "Methods"] (not a CONSORT item)

X26-i) Comment on ethics committee approval

	1	2	3	4	5	
subitem not at all important	(selected)	○	○	○	○	essential

Clear selection

Does your paper address subitem X26-i?

Copy and paste relevant sections from the manuscript (include quotes in quotation marks "like this" to indicate direct quotes from your manuscript), or elaborate on this item by providing additional information not in the ms, or briefly explain why the item is not applicable/relevant for your study

Yes. From Ethics Approval: “This study was approved by the Medical University of South Carolina’s Institutional Review Board for the Protection of Human Subjects.”

x26-ii) Outline informed consent procedures

Outline informed consent procedures e.g., if consent was obtained offline or online (how? Checkbox, etc.?), and what information was provided (see 4a-ii). See [6] for some items to be included in informed consent documents.

	1	2	3	4	5	
subitem not at all important	(●)	○	○	○	○	essential

Clear selection

Does your paper address subitem X26-ii?

Copy and paste relevant sections from the manuscript (include quotes in quotation marks "like this" to indicate direct quotes from your manuscript), or elaborate on this item by providing additional information not in the ms, or briefly explain why the item is not applicable/relevant for your study

Informed consent was obtained online prior to enrollment. From Methods: “All participants reviewed an online informed consent waiver prior to enrollment.” Consent was documented by the participant proceeding past the online consent document and completing the demographics form. Because no more than minimal risk was involved and no protected health information was collected, an IRB-approved waiver of documented signed consent was used.

X26-iii) Safety and security procedures

Safety and security procedures, incl. privacy considerations, and any steps taken to reduce the likelihood or detection of harm (e.g., education and training, availability of a hotline)

	1	2	3	4	5	
subitem not at all important	(●)	( )	( )	( )	( )	essential

Clear selection

Does your paper address subitem X26-iii?

Copy and paste relevant sections from the manuscript (include quotes in quotation marks "like this" to indicate direct quotes from your manuscript), or elaborate on this item by providing additional information not in the ms, or briefly explain why the item is not applicable/relevant for your study

From Methods: “No participant identifying information was shared with the AI model or retained by the AI provider.” Case session content was processed via the OpenAI API as a stateless call; the platform did not transmit participant names, demographics, or any identifiers into the API request. Standard web security (HTTPS) was used for the study website. Because the intervention involved no medical care or procedures and the content was educational, no clinical safety procedures were required.

RESULTS

13a) For each group, the numbers of participants who were randomly assigned, received intended treatment, and were analysed for the primary outcome

NPT: The number of care providers or centers performing the intervention in each group and the number of patients treated by each care provider in each center

Does your paper address CONSORT subitem 13a? *

Copy and paste relevant sections from the manuscript (include quotes in quotation marks "like this" to indicate direct quotes from your manuscript), or elaborate on this item by providing additional information not in the ms, or briefly explain why the item is not applicable/relevant for your study

Yes. From Results: “A total of 74 participants enrolled, and 58 completed all study components.” Of completers, 27 (47%) were in the text-based case group and 31 (53%) were in the interactive case group. All 58 completers received their assigned intervention and were analyzed in the complete-case analysis. Non-completers (n=16) dropped out before the case-study portion; no outcome data were available for this group.

13b) For each group, losses and exclusions after randomisation, together with reasons

Does your paper address CONSORT subitem 13b? (NOTE: Preferably, this is shown in a CONSORT flow diagram) *

Copy and paste relevant sections from the manuscript (include quotes in quotation marks "like this" to indicate direct quotes from your manuscript), or elaborate on this item by providing additional information not in the ms, or briefly explain why the item is not applicable/relevant for your study

Yes. From the Attrition section: “Reasons for discontinuing participation were unknown. All 16 participants who dropped out of the study did so before finishing the video training and did not start the case study portion of the study.” Of the 16 non-completers, 7 had been allocated to the text-based arm and 9 to the interactive arm (no significant between-arm difference in attrition: ChiSq=0.47, p=.49).

13b-i) Attrition diagram

Strongly recommended: An attrition diagram (e.g., proportion of participants still logging in or using the intervention/comparator in each group plotted over time, similar to a survival curve) or other figures or tables demonstrating usage/dose/engagement.

	1	2	3	4	5	
subitem not at all important	◉	○	○	○	○	essential

Clear selection

Does your paper address subitem 13b-i?

Copy and paste relevant sections from the manuscript or cite the figure number if applicable (include quotes in quotation marks "like this" to indicate direct quotes from your manuscript), or elaborate on this item by providing additional information not in the ms, or briefly explain why the item is not applicable/relevant for your study

Because the intervention was delivered in a single session, a time-course attrition curve is not applicable.

14a) Dates defining the periods of recruitment and follow-up

Does your paper address CONSORT subitem 14a? *

Copy and paste relevant sections from the manuscript (include quotes in quotation marks "like this" to indicate direct quotes from your manuscript), or elaborate on this item by providing additional information not in the ms, or briefly explain why the item is not applicable/relevant for your study

Yes. From Methods: “Study enrollment opened on April 1, 2025, and closed on July 31, 2025.” Because the study was a single-session design, “follow-up” is equivalent to the post-intervention assessments conducted within the same session as the pre-assessment and case work. No separate post-session follow-up period was implemented.

14a-i) Indicate if critical “secular events” fell into the study period

Indicate if critical “secular events” fell into the study period, e.g., significant changes in Internet resources available or “changes in computer hardware or Internet delivery resources”

	1	2	3	4	5	
subitem not at all important	(●)	( )	( )	( )	( )	essential

Clear selection

Does your paper address subitem 14a-i?

Copy and paste relevant sections from the manuscript (include quotes in quotation marks "like this" to indicate direct quotes from your manuscript), or elaborate on this item by providing additional information not in the ms, or briefly explain why the item is not applicable/relevant for your study

No critical external events (e.g., platform outages, changes in Internet infrastructure, institutional policy changes) affected the study during the April–July 2025 enrollment period.

14b) Why the trial ended or was stopped (early)

Does your paper address CONSORT subitem 14b? *

Copy and paste relevant sections from the manuscript (include quotes in quotation marks "like this" to indicate direct quotes from your manuscript), or elaborate on this item by providing additional information not in the ms, or briefly explain why the item is not applicable/relevant for your study

The trial ended as planned at the close of the enrollment window on July 31, 2025, corresponding to the end of the spring semester recruiting period. The trial was not stopped early.

15) A table showing baseline demographic and clinical characteristics for each group

NPT: When applicable, a description of care providers (case volume, qualification, expertise, etc.) and centers (volume) in each group

Does your paper address CONSORT subitem 15? *

Copy and paste relevant sections from the manuscript (include quotes in quotation marks "like this" to indicate direct quotes from your manuscript), or elaborate on this item by providing additional information not in the ms, or briefly explain why the item is not applicable/relevant for your study

Yes. Table 1 reports baseline characteristics by arm (text-based cases vs. AI-powered cases) including sex, age, race, ethnicity, degree program, completion status, and mean pre-test knowledge score. The manuscript states: “No significant differences were found in sample sizes by sex, race, ethnicity, degree program, group assignment, or study completion status.”

15-i) Report demographics associated with digital divide issues

In ehealth trials it is particularly important to report demographics associated with digital divide issues, such as age, education, gender, social-economic status, computer/Internet/ehealth literacy of the participants, if known.

	1	2	3	4	5	
subitem not at all important	◉	○	○	○	○	essential

Clear selection

Does your paper address subitem 15-i? *

Copy and paste relevant sections from the manuscript (include quotes in quotation marks "like this" to indicate direct quotes from your manuscript), or elaborate on this item by providing additional information not in the ms, or briefly explain why the item is not applicable/relevant for your study

Table 1 reports age, sex, race, ethnicity, and degree program. Because all participants were active health professional students at a US academic health sciences university, the sample represents a population with high baseline computer and internet literacy by design; explicit digital-literacy variables were not collected. Socioeconomic status was also not collected.

16) For each group, number of participants (denominator) included in each analysis and whether the analysis was by original assigned groups

16-i) Report multiple “denominators” and provide definitions

Report multiple “denominators” and provide definitions: Report N’s (and effect sizes) “across a range of study participation [and use] thresholds” [1], e.g., N exposed, N consented, N used more than x times, N used more than y weeks, N participants “used” the intervention/comparator at specific pre-defined time points of interest (in absolute and relative numbers per group). Always clearly define “use” of the intervention.

	1	2	3	4	5	
subitem not at all important	(selected)					essential

Clear selection

Does your paper address subitem 16-i? *

Copy and paste relevant sections from the manuscript (include quotes in quotation marks "like this" to indicate direct quotes from your manuscript), or elaborate on this item by providing additional information not in the ms, or briefly explain why the item is not applicable/relevant for your study

Denominators: N=74 enrolled; N=58 completed all components and were analyzed (27 text-based, 31 interactive). Because the study was a single-session design with no graded exposure, traditional multi-threshold denominators (e.g., “used the app at least X times”) are not applicable. “Use” was defined as completion of all study components in one sitting.

16-ii) Primary analysis should be intent-to-treat

Primary analysis should be intent-to-treat, secondary analyses could include comparing only “users”, with the appropriate caveats that this is no longer a randomized sample (see 18-i).

	1	2	3	4	5	
subitem not at all important	(selected)	○	○	○	○	essential

Clear selection

Does your paper address subitem 16-ii?

Copy and paste relevant sections from the manuscript (include quotes in quotation marks "like this" to indicate direct quotes from your manuscript), or elaborate on this item by providing additional information not in the ms, or briefly explain why the item is not applicable/relevant for your study

The primary analysis was a complete-case analysis rather than a formal intent-to-treat analysis, as the 16 non-completers dropped out before any outcome data were collected (before the case-study portion). This is acknowledged as a limitation. Baseline balance checks (Table 1) confirmed no significant differences in demographics between completers and non-completers.

17a) For each primary and secondary outcome, results for each group, and the estimated effect size and its precision (such as 95% confidence interval)

Does your paper address CONSORT subitem 17a? *

Copy and paste relevant sections from the manuscript (include quotes in quotation marks "like this" to indicate direct quotes from your manuscript), or elaborate on this item by providing additional information not in the ms, or briefly explain why the item is not applicable/relevant for your study

Yes. Knowledge gains: significant time×group interaction, F(1,56)=4.39, P=.041, with post-test between-group difference P=.029. Case discussion quiz accuracy: interactive mean 0.78 (SD 0.15) vs. text mean 0.65 (SD 0.12); t(62)=4.04, P<.001; estimated 20.7% advantage for the interactive condition. Engagement: 13 of 17 items significantly favored the interactive arm (P<.05); see Table 2 for all 17 items with means, SDs, t-values, df, and p-values for each comparison. Figure 2A shows pre-post change by group; Figure 2B shows case discussion quiz mean with 95% CI by group; Figure 3 shows 95% CIs for five key engagement items.

17a-i) Presentation of process outcomes such as metrics of use and intensity of use

In addition to primary/secondary (clinical) outcomes, the presentation of process outcomes such as metrics of use and intensity of use (dose, exposure) and their operational definitions is critical. This does not only refer to metrics of attrition (13-b) (often a binary variable), but also to more continuous exposure metrics such as “average session length”. These must be accompanied by a technical description how a metric like a “session” is defined (e.g., timeout after idle time) [1] (report under item 6a).

	1	2	3	4	5	
subitem not at all important	◉	○	○	○	○	essential

Clear selection

Does your paper address subitem 17a-i?

Copy and paste relevant sections from the manuscript (include quotes in quotation marks "like this" to indicate direct quotes from your manuscript), or elaborate on this item by providing additional information not in the ms, or briefly explain why the item is not applicable/relevant for your study

Process metrics (time-on-task, number of messages exchanged per interview, session length) were not analyzed for this report. This is explicitly acknowledged as a limitation: “time-on-task was not measured…Future studies should incorporate digital trace data or controlled time conditions to clarify these relationships.”

17b) For binary outcomes, presentation of both absolute and relative effect sizes is recommended

Does your paper address CONSORT subitem 17b? *

Copy and paste relevant sections from the manuscript (include quotes in quotation marks "like this" to indicate direct quotes from your manuscript), or elaborate on this item by providing additional information not in the ms, or briefly explain why the item is not applicable/relevant for your study

The primary outcomes were continuous (knowledge percent correct; engagement Likert scores) or a bounded proportion (case discussion accuracy). The case discussion accuracy is reported in both absolute terms (mean 0.78 vs. 0.65) and as a relative difference (20.7% advantage). No strictly binary (yes/no) outcomes were analyzed as primary endpoints.

18) Results of any other analyses performed, including subgroup analyses and adjusted analyses, distinguishing pre-specified from exploratory

Does your paper address CONSORT subitem 18? *

Copy and paste relevant sections from the manuscript (include quotes in quotation marks "like this" to indicate direct quotes from your manuscript), or elaborate on this item by providing additional information not in the ms, or briefly explain why the item is not applicable/relevant for your study

Baseline balance checks (Table 1) comparing demographics by arm and by completion status are reported as pre-specified balance assessments, not as hypothesis tests. No subgroup analyses (e.g., by degree program or sex) were performed. No adjusted analyses were conducted.

18-i) Subgroup analysis of comparing only users

A subgroup analysis of comparing only users is not uncommon in ehealth trials, but if done, it must be stressed that this is a self-selected sample and no longer an unbiased sample from a randomized trial (see 16-iii).

	1	2	3	4	5	
subitem not at all important	◉	○	○	○	○	essential

Clear selection

Does your paper address subitem 18-i?

Copy and paste relevant sections from the manuscript (include quotes in quotation marks "like this" to indicate direct quotes from your manuscript), or elaborate on this item by providing additional information not in the ms, or briefly explain why the item is not applicable/relevant for your study

Not applicable. All analyzed participants (N=58) completed all study components; there is no “users vs. non-users” subgroup within the analyzed sample.

19) All important harms or unintended effects in each group
(for specific guidance see CONSORT for harms)

Does your paper address CONSORT subitem 19? *

Copy and paste relevant sections from the manuscript (include quotes in quotation marks "like this" to indicate direct quotes from your manuscript), or elaborate on this item by providing additional information not in the ms, or briefly explain why the item is not applicable/relevant for your study

No harms or adverse events were observed or reported. The intervention was an educational activity delivered online; no clinical procedures, treatments, or decisions affecting participant health were involved. Unintended effects on learning (e.g., negative misconceptions induced by AI-generated content) were not specifically measured beyond the pre/post knowledge assessment, on which the interactive arm showed equal or greater gains than the control arm.

19-i) Include privacy breaches, technical problems

Include privacy breaches, technical problems. This does not only include physical “harm” to participants, but also incidents such as perceived or real privacy breaches [1], technical problems, and other unexpected/unintended incidents. “Unintended effects” also includes unintended positive effects [2].

	1	2	3	4	5	
subitem not at all important	(selected)	○	○	○	○	essential

Clear selection

Does your paper address subitem 19-i?

Copy and paste relevant sections from the manuscript (include quotes in quotation marks "like this" to indicate direct quotes from your manuscript), or elaborate on this item by providing additional information not in the ms, or briefly explain why the item is not applicable/relevant for your study

No technical problems, harm to participants or privacy breaches.

19-ii) Include qualitative feedback from participants or observations from staff/researchers

Include qualitative feedback from participants or observations from staff/researchers, if available, on strengths and shortcomings of the application, especially if they point to unintended/unexpected effects or uses. This includes (if available) reasons for why people did or did not use the application as intended by the developers.

	1	2	3	4	5	
subitem not at all important	(●)	○	○	○	○	essential

Clear selection

Does your paper address subitem 19-ii?

Copy and paste relevant sections from the manuscript (include quotes in quotation marks "like this" to indicate direct quotes from your manuscript), or elaborate on this item by providing additional information not in the ms, or briefly explain why the item is not applicable/relevant for your study

No qualitative student feedback about the platform is provided in the manuscript. Only quantitative data about utility, value and satisfaction.

DISCUSSION

22) Interpretation consistent with results, balancing benefits and harms, and considering other relevant evidence

NPT: In addition, take into account the choice of the comparator, lack of or partial blinding, and unequal expertise of care providers or centers in each group

22-i) Restate study questions and summarize the answers suggested by the data, starting with primary outcomes and process outcomes (use)

Restate study questions and summarize the answers suggested by the data, starting with primary outcomes and process outcomes (use).

	1	2	3	4	5	
subitem not at all important	(●)	( )	( )	( )	( )	essential

Clear selection

Does your paper address subitem 22-i? *

Copy and paste relevant sections from the manuscript (include quotes in quotation marks "like this" to indicate direct quotes from your manuscript), or elaborate on this item by providing additional information not in the ms, or briefly explain why the item is not applicable/relevant for your study

The hypothesis that interactive virtual patient cases would produce greater learning outcomes than written cases was supported across all three pre-specified domains (knowledge, case discussion accuracy, engagement). The manuscript also explicitly notes what the study does NOT establish: “This study cannot isolate the independent contribution of AI or time-on-task from the broader effects of interactivity.”

22-ii) Highlight unanswered new questions, suggest future research

Highlight unanswered new questions, suggest future research.

	1	2	3	4	5	
subitem not at all important	(•)	( )	( )	( )	( )	essential

Clear selection

Does your paper address subitem 22-ii?

Copy and paste relevant sections from the manuscript (include quotes in quotation marks "like this" to indicate direct quotes from your manuscript), or elaborate on this item by providing additional information not in the ms, or briefly explain why the item is not applicable/relevant for your study

Discussed in the Comparison With Prior Work section, which situates the findings in the simulation-based education and active-learning literature (references 5–6, 10–15) and notes both alignment with and extension of prior work.

20) Trial limitations, addressing sources of potential bias, imprecision, and, if relevant, multiplicity of analyses

20-i) Typical limitations in ehealth trials

Typical limitations in ehealth trials: Participants in ehealth trials are rarely blinded. Ehealth trials often look at a multiplicity of outcomes, increasing risk for a Type I error. Discuss biases due to non-use of the intervention/usability issues, biases through informed consent procedures, unexpected events.

	1	2	3	4	5	
subitem not at all important	◉	○	○	○	○	essential

Clear selection

Does your paper address subitem 20-i? *

Copy and paste relevant sections from the manuscript (include quotes in quotation marks "like this" to indicate direct quotes from your manuscript), or elaborate on this item by providing additional information not in the ms, or briefly explain why the item is not applicable/relevant for your study

The manuscript discusses limitations characteristic of web-based educational RCTs: voluntary self-selection that may favor technology-comfortable learners; short assessment windows that may not capture longer-term retention; single-session designs that cannot evaluate durability; and the inherent confound between the novelty of interactive formats and their pedagogical effect (novelty effect).

21) Generalisability (external validity, applicability) of the trial findings

NPT: External validity of the trial findings according to the intervention, comparators, patients, and care providers or centers involved in the trial

21-i) Generalizability to other populations

Generalizability to other populations: In particular, discuss generalizability to a general Internet population, outside of a RCT setting, and general patient population, including applicability of the study results for other organizations

	1	2	3	4	5	
subitem not at all important	(•)	( )	( )	( )	( )	essential

Clear selection

Does your paper address subitem 21-i?

Copy and paste relevant sections from the manuscript (include quotes in quotation marks "like this" to indicate direct quotes from your manuscript), or elaborate on this item by providing additional information not in the ms, or briefly explain why the item is not applicable/relevant for your study

Addressed in the passage cited under item 21. Specific risk factors for reduced generalizability are identified: single-site sample, voluntary enrollment, and lack of diversity in curricular structures.

21-ii) Discuss if there were elements in the RCT that would be different in a routine application setting

Discuss if there were elements in the RCT that would be different in a routine application setting (e.g., prompts/reminders, more human involvement, training sessions or other co-interventions) and what impact the omission of these elements could have on use, adoption, or outcomes if the intervention is applied outside of a RCT setting.

	1	2	3	4	5	
subitem not at all important	(●)	○	○	○	○	essential

Clear selection

Does your paper address subitem 21-ii?

Copy and paste relevant sections from the manuscript (include quotes in quotation marks "like this" to indicate direct quotes from your manuscript), or elaborate on this item by providing additional information not in the ms, or briefly explain why the item is not applicable/relevant for your study

Addressed in the Future Directions section: “Online virtual patients can deliver standardized yet adaptive encounters without the cost of simulation labs or standardized patient programs. They also provide opportunities for inclusive training through exposure to varied patient backgrounds, communication styles, and clinical complexities.” Routine-use considerations including human involvement and scalability are discussed; the AI model parameters needed to reproduce the behavior are documented in the AI Model Information section.

OTHER INFORMATION

23) Registration number and name of trial registry

Does your paper address CONSORT subitem 23? *

Copy and paste relevant sections from the manuscript (include quotes in quotation marks "like this" to indicate direct quotes from your manuscript), or elaborate on this item by providing additional information not in the ms, or briefly explain why the item is not applicable/relevant for your study

“Trial Registration: Not applicable (educational intervention; IRB-approved by the Medical University of South Carolina Institutional Review Board for the Protection of Human Subjects).” The study was an educational RCT not evaluating a medical intervention and was not prospectively registered on ClinicalTrials.gov or an equivalent registry.

24) Where the full trial protocol can be accessed, if available

Does your paper address CONSORT subitem 24? *

Cite a Multimedia Appendix, other reference, or copy and paste relevant sections from the manuscript (include quotes in quotation marks "like this" to indicate direct quotes from your manuscript), or elaborate on this item by providing additional information not in the ms, or briefly explain why the item is not applicable/relevant for your study

The full protocol is not separately published. Methods, case content (Multimedia Appendix 2), the knowledge test (Multimedia Appendix 1), and the learner engagement survey (Multimedia Appendix 3) together document the complete protocol.

25) Sources of funding and other support (such as supply of drugs), role of funders

Does your paper address CONSORT subitem 25? *

Copy and paste relevant sections from the manuscript (include quotes in quotation marks "like this" to indicate direct quotes from your manuscript), or elaborate on this item by providing additional information not in the ms, or briefly explain why the item is not applicable/relevant for your study

Yes. From Acknowledgments: “This work was funded by National Institutes of Health (NIH) Small Business Technology Transfer (STTR) Grant 1R41DA059281-01A1. The funders had no role in study design, data collection and analysis, decision to publish, or preparation of the manuscript.” Conflicts of interest are disclosed: “JJB and DH are employed by the Medical University of South Carolina (MUSC) and are founders of Palmetto Innovative Education LLC, doing business as Figment Learning Labs — the company formed to commercialize the Figment Learning Labs software developed at MUSC through NIH funding.”

X27) Conflicts of Interest (not a CONSORT item)

X27-i) State the relation of the study team towards the system being evaluated

In addition to the usual declaration of interests (financial or otherwise), also state the relation of the study team towards the system being evaluated, i.e., state if the authors/evaluators are distinct from or identical with the developers/sponsors of the intervention.

	1	2	3	4	5	
subitem not at all important	◉	○	○	○	○	essential

Clear selection

Does your paper address subitem X27-i?

Copy and paste relevant sections from the manuscript (include quotes in quotation marks "like this" to indicate direct quotes from your manuscript), or elaborate on this item by providing additional information not in the ms, or briefly explain why the item is not applicable/relevant for your study

The sample was predominantly female (72%), predominantly white (72%), and drawn from one US academic health sciences university. Distribution across degree programs was skewed toward allied health (59%), with smaller representation from medicine, pharmacy, dentistry, graduate studies, and nursing. This profile is broadly consistent with interprofessional course enrollments at the host institution but limits direct extrapolation to other institutions, learner populations, and content domains, as explicitly discussed in Limitations.

About the CONSORT EHEALTH checklist

As a result of using this checklist, did you make changes in your manuscript? *

- ○ yes, major changes
- ○ yes, minor changes
- ● no

What were the most important changes you made as a result of using this checklist?

Your answer

How much time did you spend on going through the checklist INCLUDING making changes in your manuscript *

It took 3 hours to complete this form.

As a result of using this checklist, do you think your manuscript has improved? *

- ○ yes
- ◉ no
- ○ Other:

Would you like to become involved in the CONSORT EHEALTH group?

This would involve for example becoming involved in participating in a workshop and writing an "Explanation and Elaboration" document

- ○ yes
- ◉ no
- ○ Other:

Clear selection

Any other comments or questions on CONSORT EHEALTH

Your answer

STOP - Save this form as PDF before you click submit

To generate a record that you filled in this form, we recommend to generate a PDF of this page (on a Mac, simply select "print" and then select "print as PDF") before you submit it.

When you submit your (revised) paper to JMIR, please upload the PDF as supplementary file.

Don't worry if some text in the textboxes is cut off, as we still have the complete information in our database. Thank you!

Final step: Click submit !

Click submit so we have your answers in our database!

Submit

Clear form

Never submit passwords through Google Forms.

This content is neither created nor endorsed by Google. - Contact form owner - Terms of Service - Privacy Policy

Does this form look suspicious? Report

Google Forms
