# Supplementary material for: Knowledge Acquisition, Case Discussion, and Engagement in Health Professional Students Using Interactive Virtual Patient Cases Versus Written Case Studies: Randomized Controlled Trial
Source: JMIR Nurs. 2026 Sep 23;9:e98700. doi: 10.2196/98700 (PMC13600594; doi:10.2196/98700)
Supplement: Supplementary file 2 [file nursing-v9-e98700-s001.docx]

**Pre- and Post-training Knowledge Test**

1. Hip and knee pain associated with arthritis is best categorized as:

A) Nociceptive

B) Neuropathic

C) Acute

D) Chronic

2. Burning pain originating in the lower back that shoots down the upper legs is best described as:

A) Nociceptive

B) Neuropathic

C) Acute

D) Chronic

3. Chronic pain is pain that persists for at least:

A. 1-month

B. 3-months

C. 8-months

D. 12-months

4. Nonsteroidal Anti-Inflammatory Drugs are effective for managing (Check all that apply):

[] Chronic pain

[] Acute pain

[] Musculoskeletal injuries

[] Neuropathic pain

[] Phantom-limb pain

[] Post-surgical pain

5. Non-Pharmacologic pain treatments are significantly less effective than medication-based pain management strategies.

[] True

[] False

6. Opioids are a reasonable first-line pain management option for severe chronic pain.

[] True

[] False

7. Black patients are less likely to receive opioids for pain and receive lower doses than white patients.

[] True

[] False

8. Pharmacies in non-white neighborhoods are less likely to have a reasonable range of opioid medications available.

[] True

[] False

**Case Studies**

**Case#1:**

Name: Mark Stevenson

Age: 42

Sex: Male

Race: African American

Ethnicity: Non-Hispanic

Height: 5’7”

Weight: 185 pounds

Presenting Problem: Low back pain

Symptoms: Aching dull pain in lower back, with burning, electrical pain shooting down the left leg, pain is rated 7/10 on average, depressed mood, sleep disturbance

History of Present Illness: Pain started about 10 years ago after falling off a ladder at work. Tried physical therapy, medications, spinal cord stimulator. All produced limited benefit. Patient had an Emergency Room visit about 6 months ago related to pain, but the records are not available here.

Social History: Patient is married for 25 years. Has 2 adult children. Completed high school. Worked as a house painter until injuring back 10 years ago after a fall from a ladder. Currently receiving disability for back pain.

Family Medical History: Patient has a 10-year history of type 2 diabetes. Father and mother both have diabetes. Mother has high blood pressure. Father has depression.

Visit Info: Patient reports that he has been prescribed opioids in the past for his back pain with good benefit and would like to explore re-starting opioid therapy for his pain. He reports that they are the only thing that work for him. He is forthcoming about pain symptoms but denied depression symptoms. His previous provider discontinued opioids a few months ago, but the reasons are not clear and no records are available at the time of the interview. The patient was taken to the Emergency Room about 6 months ago for difficulty breathing, lethargy and losing consciousness, but the patient reports that he does not remember what the findings were. He says he has been doing fine ever since, and the problems have not come back.

**Case #2:**

Name: Sarah Jones

Age: 36

Sex: Female

Race: African American

Ethnicity: Non-Hispanic

Height: 5’7”

Weight: 120 pounds

Presenting Problem: Bilateral knee pain

Symptoms: Pain in both knees, right knee pain (7/10) is greater than left (4/10). Pain is described as aching and throbbing. Patient denies depression or anxiety but noted that the pain interferes with her sleep (currently only getting about 4-5 hours per night due to pain-related waking).

History of Present Illness: Patient was a gymnast from the age of 6 through 18. Her primary events were balance-beam and vaulting. She believes her heavy involvement in the activities slowly took a toll on her knees over many many years. Pain started to become noticeable and problematic about 8 months ago and has progressively gotten worse. Pain is exacerbated by walking and standing. Elevating her feet helps only a little, but ibuprofen provides moderate relief. Patient used to enjoy running for exercise but had to stop about 6 months ago due to pain.

Social History: Patient is married for 10 years. Has 1 male child age 8. Completed college with a degree in accounting. Has worked at the same tax-accounting firm for 12 years. Was an accomplished gymnast with Olympic potential, tried-out, but did not make the Olympic team. Stopped gymnastics at the age of 18 and went to college.

Family Medical History: Patient denied history of any chronic medical conditions including mental health conditions for herself and for her family.

Visit Info: Patient is forthcoming and open about her symptoms, but is slightly agitated because of her pain. She misses her gymnastics days, but is conflicted because she is certain that her physical activity caused her current pain and dysfunction. She does not like to take medications, but is desperately seeking relief and is feeling worn-out because of her lack of sleep, so she is willing to try anything that might help. She is annoyed by having to complete clinic paperwork, and just wants help. There is no evidence of depression or anxiety, but she seems to be getting more and more concerned and feeling hopeless about her current pain experience.

**Case #3:**

Name: David Hinson

Age: 49

Sex: Male

Race: Caucasian

Ethnicity: Non-Hispanic

Height: 5’11”

Weight: 185 pounds

Presenting Problem: Shoulder and arm pain

Symptoms: Pain in left shoulder that radiates down the arm to the hand. Pain is rated as 8/10 and is described as burning and shooting. Lifting the arm above the shoulder exacerbates the pain.

History of Present Illness: Patient fell down the stairs a little over 2 months ago and extended his left arm to catch himself. He landed on it and experienced severe pain. He went to the emergency room but X-rays showed no significant tissue or bone damage. Pain got worse over the next several weeks. He has tried Tylenol and ice packs with no benefit. He went to his primary care doctor who referred him here for a more in-depth pain assessment.

Social History: Patient is divorced for 2 years. Has 3 adult children. Completed a masters degree in graphic design and worked for a design company most of his life, before switching to freelance design work about 4 years ago. He experienced mild depression when his father died about 4 years ago and still has some occasional bouts of bereavement and grief.

Family Medical History: Patient has a family history of depression and hypertension (father) but denied any chronic medical illnesses for himself.

Visit Info: Patient is very cooperative during the interview and is willing to work hard to find a solution to his shoulder and arm pain. He is engaging and kind. He is open to all treatment recommendations including non-pharmacologic treatment as well as referrals to specialty care for further evaluation.

**Learner Engagement Survey**

Participant Feedback on Online Case-Study Training

Instructions: Please indicate how much you agree or disagree with each of the following statements using the 5-point scale below.

**1 = Strongly Disagree	2 = Disagree	3 = Neither Agree nor Disagree	4 = Agree	5 = Strongly Agree**

| **Please rate your agreement with each statement.** | **1**  **Strongly**  **Disagree** | **2**  **Disagree** | **3**  **Neutral** | **4**  **Agree** | **5**  **Strongly**  **Agree** |
| --- | --- | --- | --- | --- | --- |
| **1.** The online training interface was easy to use. | ○ | ○ | ○ | ○ | ○ |
| **2.** The instructions for completing the online training were clear and understandable. | ○ | ○ | ○ | ○ | ○ |
| **3.** The online training platform is technologically sophisticated. | ○ | ○ | ○ | ○ | ○ |
| **4.** The online training platform is visually appealing. | ○ | ○ | ○ | ○ | ○ |
| **5.** The case-studies portion of the learning activity met the learning objectives. | ○ | ○ | ○ | ○ | ○ |
| **6.** The case-studies felt like ‘real world’ scenarios. | ○ | ○ | ○ | ○ | ○ |
| **7.** The case-studies portion of the training made learning more interesting and fun. | ○ | ○ | ○ | ○ | ○ |
| **8.** I felt that I learned something from the case-studies. | ○ | ○ | ○ | ○ | ○ |
| **9.** I learned techniques and information during this training that will help me in the real world. | ○ | ○ | ○ | ○ | ○ |
| **10.** The learning activity helped me practice clinical decision-making skills. | ○ | ○ | ○ | ○ | ○ |
| **11.** The case-studies enhanced my ability to apply theoretical knowledge in a practical scenario. | ○ | ○ | ○ | ○ | ○ |
| **12.** The case-studies felt realistic. | ○ | ○ | ○ | ○ | ○ |
| **13.** This training technology increased my engagement with the learning material. | ○ | ○ | ○ | ○ | ○ |
| **14.** I am motivated to use activities like online case-studies to enhance my learning experience. | ○ | ○ | ○ | ○ | ○ |
| **15.** This training activity made learning more enjoyable. | ○ | ○ | ○ | ○ | ○ |
| **16.** I am satisfied with this learning activity. | ○ | ○ | ○ | ○ | ○ |
| **17.** I would recommend this learning activity to my peers. | ○ | ○ | ○ | ○ | ○ |
